# Development of a Modular Ribonucleoprotein Complex as a General Strategy to Deliver RNAi Therapeutics

**DOI:** 10.1002/adhm.202503281

**Published:** 2025-07-23

**Authors:** Nok Yin Tam, Xiaoqi Wang, Grace Chung Yan Chan, Wai Po Kong, Wai Yin Chau, Xiuqiong Fu, Kwok‐Yin Wong, Hong Lok Lung, Zhi‐Ling Yu, Wei Shen Aik

**Affiliations:** ^1^ Department of Chemistry Hong Kong Baptist University Kowloon Tong Kowloon Hong Kong SAR China; ^2^ School of Chinese Medicine Hong Kong Baptist University Kowloon Tong Kowloon Hong Kong SAR China; ^3^ State Key Laboratory of Chemical Biology and Drug Discovery Department of Applied Biology and Chemical Technology The Hong Kong Polytechnic University Kowloon Hong Kong SAR China

**Keywords:** KRAS, protein‐based delivery agent, ribonucleoprotein complex, RNAi therapeutics, siRNA delivery

## Abstract

RNAi therapeutics can potentially address undruggable diseases. However, their full potential is stymied by delivery challenges. An siRNA delivery platform by remodeling the human U4 small nuclear ribonucleoprotein complex (snRNP) is developed. This remodeled protein‐siRNA complex (SmiRNP) serves as a biocompatible, modular, and customizable platform for attaching desired functional modules to overcome delivery hurdles. Here, it is demonstrated that the SmiRNP complex, by using siRNA against *KRAS* as a proof of principle, can deliver the siRNA into cells and protect it from nuclease degradation, allowing the siRNA to knockdown *KRAS* mRNA and protein levels, reduce cancer cell viability, and suppress tumor growth in vivo. This general strategy allows many more modular combinations and would enable the delivery of a wide spectrum of RNAi therapeutics.

## Graphical abstract

A modular and customizable RNAi therapeutics delivery platform composed of a pseudosymmetric human RNA binding protein‐siRNA complex (SmiRNP) is developed. By fusing a targeting module and an endosomal escape peptide onto the delivery platform, SmiRNP successfully delivered siRNA to silence the *KRAS* oncogene in a colorectal carcinoma cell line, leading to cancer cell growth suppression in vitro and in vivo.

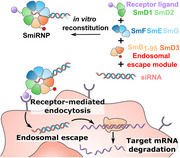

## 1 Introduction

RNA interference (RNAi) therapeutics are a type of gene therapy that uses small interfering RNAs (siRNAs) to silence target genes. siRNAs are typically short double‐stranded RNAs (dsRNA) with 21–23 nucleotides. Once introduced into the cell, siRNAs will be processed and loaded onto the RNA‐induced silencing complex (RISC), which will cleave mRNAs complementary to the guide RNA strand, thus leading to gene silencing.^[^ ^1^ ^]^ By introducing synthetic siRNAs into cells, this gene silencing process can be exploited as gene‐therapy‐based treatments for diseases that are deemed undruggable by small‐molecule chemotherapy.^[^ ^2^ ^]^ As RNAi therapeutics function by complementary base pairing, they are highly selective. However, delivery of RNAi therapeutics to target tissues remains challenging. RNA drugs can easily be degraded by nucleases, do not readily cross the cell membrane, and might elicit an immune response.^[^ ^3^ ^]^ To date, delivery of RNAi therapeutics using lipid nanoparticles and ligand conjugations, for example GalNAc, has seen the most success, especially in targeting the liver.^[^ ^4^ ^]^ Otherwise, delivery of RNAi therapeutics to other tissues using current methods remains challenging. With our expertise in structural and chemical biology of protein‐RNA complexes, we reconstituted a remodeled ribonucleoprotein complex as an RNAi therapeutics delivery agent. Before this work, several reports described the use of proteins as siRNA delivery agents, e.g. dsRNA binding domain (dsRBD) from PKR^[^ ^5^ ^]^ and U1A RBD.^[^ ^6^ ^]^ However, these methods require additional measures for effective gene silencing, such as PEGylation, chemical conjugation, and photoactivation, limiting their potential in clinical use.^[^ ^5^, ^6^ ^]^

Here, we developed a protein‐based RNAi therapeutics delivery platform that is modular and customizable, potentially complementing existing delivery technologies in targeting extrahepatic tissues. Our siRNA delivery platform contains a heteroheptameric scaffold modified from a U‐rich small nuclear ribonucleoprotein complex (snRNP), which consists of 7 Sm proteins binding to a U‐rich consensus sequence inserted into the siRNA. We henceforth call it Sm‐siRNA ribonucleoprotein complex (SmiRNP). Each Sm protein is unique and has 2 termini (N and C) available for protein fusion. The multimeric core serves as a scaffold for fusing up to possibly 14 functional protein modules to target different tissues and cross biological hurdles. The delivery platform is largely made of human proteins for biocompatibility. As proof‐of‐concept, we demonstrate that the SmiRNP can deliver siRNA to silence *KRAS* in colorectal carcinoma cells, inducing cancer cell death in vitro and restraining tumor growth in vivo.

## 2 Results and Discussion

### 2.1 Design and Reconstitution of SmiRNP

We envisioned the human small nuclear ribonucleoprotein complexes (snRNPs) as a starting point for engineering a modular siRNA delivery agent because snRNPs contain small nuclear RNAs (snRNAs),^[^ ^7^ ^]^ which are similar in structure to siRNAs, and the core protein components are heteroheptameric. Each of the 7 subunits can act as a distinct domain for attaching functional modules through protein fusion. In mammals, there exist different versions of snRNPs (e.g., U1 snRNP, U4 snRNP, and U7 snRNP) for particular cellular functions such as pre‐mRNA splicing and 3′‐end processing,^[^ ^7^, ^8^ ^]^ suggesting that they have been customized for specialized purposes by evolution. In the snRNP cores, the snRNAs contain a U‐rich consensus sequence that is bound by 7 SmLsm proteins. The U‐rich sequences are typically flanked by RNA hairpin duplexes^[^ ^7a,d^ ^]^. For instance, the U1 and U4 snRNP core Sm proteins (SmD1, SmD2, SmG, SmE, SmF, SmD3, and SmB) form a heptameric ring by binding the U‐rich sequence on snRNA^[^ ^7a,d^ ^]^. Structures of snRNPs showed that the U‐rich motif threads through the center hole of the Sm ring.^[^ ^7^, ^9^ ^]^

**Figure 1:**
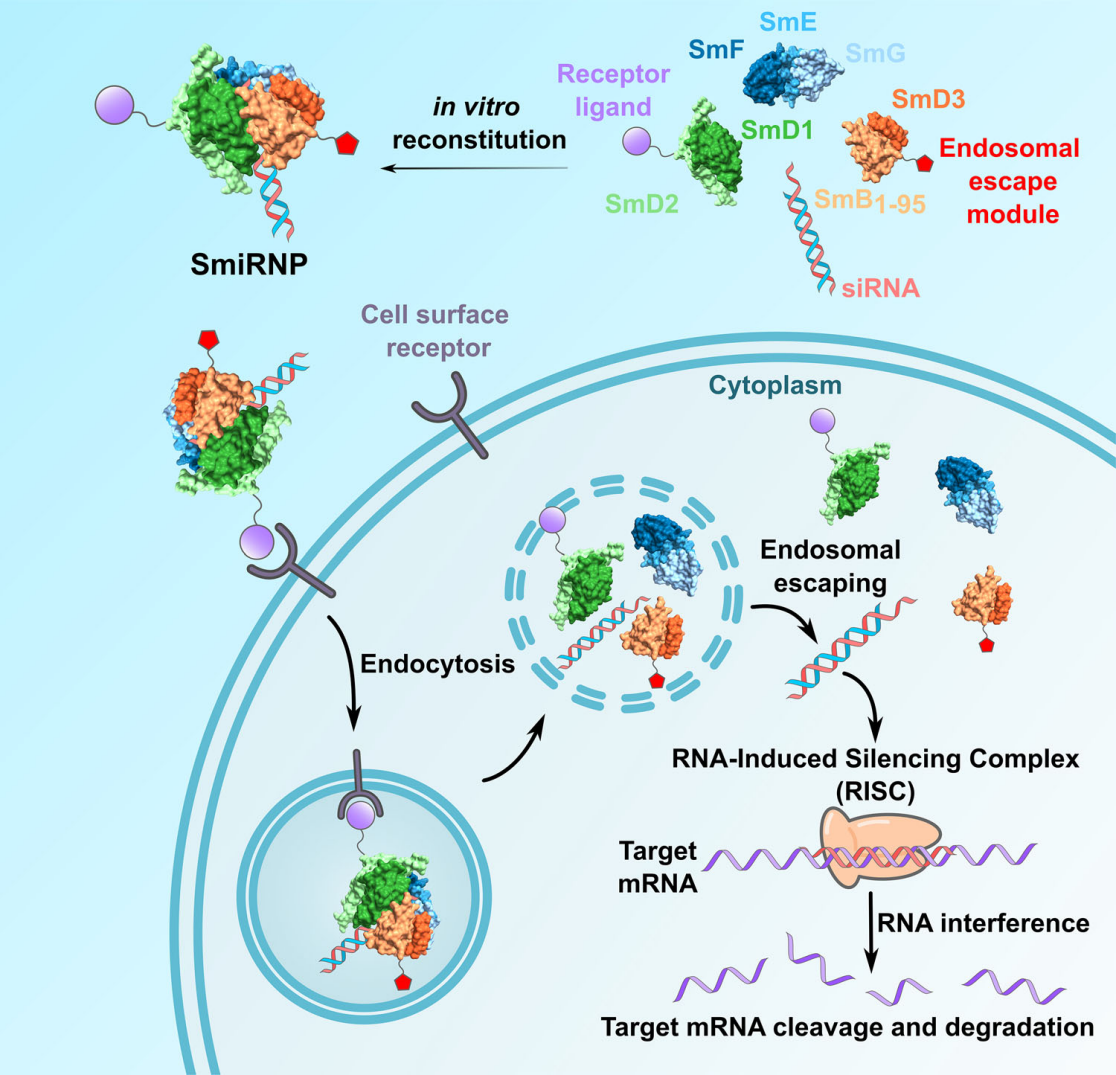
Design Concept of SmiRNP as an siRNA delivery agent. SmiRNPs are reconstituted by complexing siRNA containing a U‐rich sequence recognized by a combination of seven different Sm core proteins (SmD1/SmD2, SmD3/SmB_1‐95_, SmG/SmE/SmF) that were recombinantly purified as binary or ternary subcomplexes. Cell receptor ligands (purple circle) and endosomal escape peptides (red pentagon) can be fused to individual Sm proteins (e.g., SmD2‐EGF, SmB_1‐95_‐H5E). The reconstituted SmiRNP can then be administered in vitro or in vivo to bind cell surface receptors for receptor‐mediated endocytosis. In the cell, the endosomal escape peptide will aid in endosomal escape of the siRNA and trigger RNA interference, leading to gene silencing.

**Figure 2:**
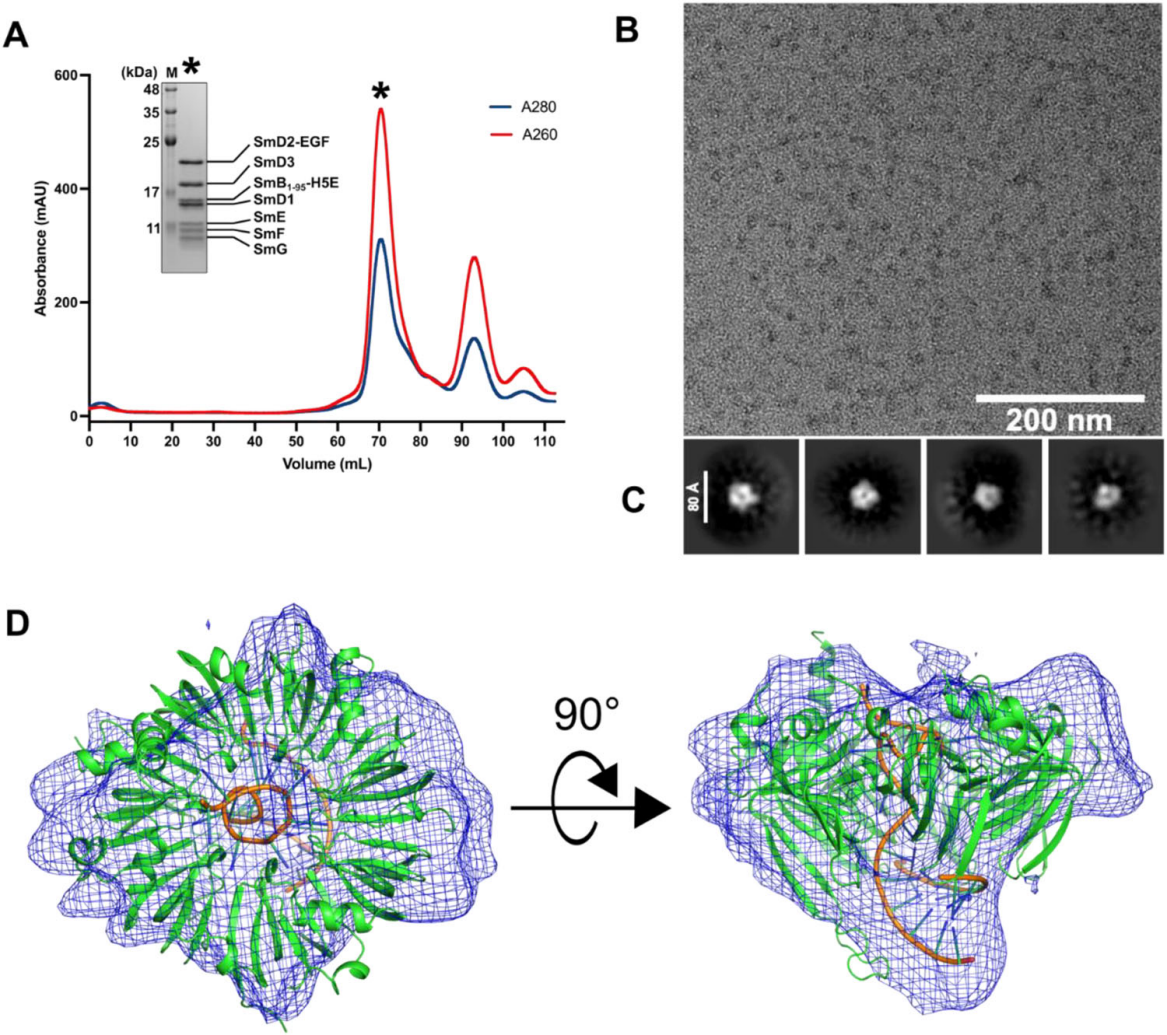
Size exclusion chromatography and negative stain electron microscopy characterization of the in vitro reconstituted SmiRNP‐EGF‐H5E. A) Size exclusion chromatography profile of the in vitro reconstituted SmiRNP‐EGF‐H5E and SDS‐PAGE analysis of the fractions from the major peak (＊). M, protein molecular weight marker; red line, UV 260 nm absorbance; blue line, UV 280 nm absorbance. B) A representative raw negative stain electron micrograph of SmiRNP‐EGF‐H5E. The scale bar indicates 200 nm. C) Representative 2D class averages of SmiRNP‐EGF‐H5E. Scale bar indicates 80 Å. D) Top (left) and side (right) views of the model of the U4 snRNP core (PDB ID 4WZJ)^[^ ^7a^ ^]^ docked into the ab initio reconstructed density volume (contour level, *σ* = 1.0) by cryoSPARC v4.4.1^[^ ^13^ ^]^ using the negative stain electron microscopy particles of SmiRNP‐EGF‐H5E. Green cartoons, Sm proteins; orange cartoon, RNA backbone; blue‐green sticks, RNA nucleosides. Parts of the RNA stem loop of the U4 snRNP model that did not fit the density were not shown. Density volume for the fused EGF ligand and H5E endosomal escape peptide was also not observed, thus not modeled. [Correction added on August 5, 2025, after first online publication: Figure 2 has been updated.]

To remodel a snRNP into a ribonucleoprotein complex that can bind siRNA (SmiRNP), we modified the U4 snRNP core (**Figure** **1**). We replaced the U4 snRNA with an siRNA that targets the *KRAS* gene and had the U4 snRNA Sm‐binding sequence (AUUUUUG) at the 5′‐end of the antisense strand (siKRAS) (Table S2, Supporting Information).^[^ ^7^, ^10^ ^]^ For cell entry by receptor‐mediated endocytosis, we fused an epidermal growth factor (EGF) to the C‐terminus of SmD2 (SmD2‐EGF). We then considered the possibility of the siRNA being trapped in the endosomes after endocytosis, resulting in degradation in the lysosomes.^[^ ^11^ ^]^ Taking advantage of the modularity of SmiRNP, we fused an endosomal escape peptide (EEP) to the SmiRNP by fusing H5E, an EEP containing the amino acid sequence HEHEHEHEH,^[^ ^12^ ^]^ to the C‐terminus of SmB residues 1–95 (SmB_1‐95_) (SmB_1‐95_‐H5E). To obtain the SmiRNP‐EGF‐H5E complex, we first recombinantly produced and purified the Sm protein subcomplexes (SmD1/SmD2‐EGF, SmG/SmE/SmF, and SmD3/SmB_1‐95_‐H5E). Subsequently, we in vitro reconstituted the Sm subcomplexes with siKRAS into a ribonucleoprotein complex (SmiRNP‐EGF‐H5E) and purified the complex by size exclusion chromatography (**Figure** **2**A). The fractions corresponding to the major peak of the size exclusion chromatography were analyzed by SDS‐PAGE. The SDS‐PAGE gel stained with Coomassie blue showed seven bands corresponding to each of the Sm proteins (Figure 2A). The sample had a UV absorption 260/280 nm ratio of 1.6, suggesting the presence of nucleic acid.

To further verify that the Sm proteins bind to the Sm sequence on the 5′‐end of the antisense strand of siKRAS, we analyzed, by size exclusion chromatography, the reconstitution of SmiRNP‐EGF‐H5E using either siKRAS or a variant of siKRAS with the Sm binding sequence 5′‐AUUUUUG‐3′ replaced with 5′‐AAAAAAG‐3′ (siKRAS (Sm^abs^)). Results from analytical size exclusion chromatography showed that the reconstitution of SmiRNP‐EGF‐H5E using siKRAS (Sm^abs^) did not result in a peak (elution volume = 13.0 mL), which corresponds to the whole SmiRNP‐EGF‐H5E complex as shown by the chromatogram of the SmiRNP‐EGF‐H5E using siKRAS containing the Sm binding sequence (Figure S2A, Supporting Information). SDS‐PAGE (Figure S2B, Supporting Information) and Urea‐PAGE (Figure S2C, Supporting Information) analyses of fractions from the peak at elution volume 13.0 mL of the SmiRNP‐EGF‐H5E sample reconstituted using siKRAS showed the presence of bands for all 7 Sm proteins and the two strands of siKRAS. The analytical size exclusion profile of the reconstitution of SmiRNP‐EGF‐H5E using siKRAS (Sm^abs^) only showed peaks corresponding to the un‐complexed proteins and siRNA, according to SDS‐PAGE and Urea‐PAGE analysis; the profiles of these peaks overlap with the profiles of the chromatograms of the protein only and siKRAS (Sm^abs^) only controls (Figure S2A–C, Supporting Information), suggesting that the larger SmiRNP‐EGF‐H5E complex did not form. These results indicate that the reconstitution of SmiRNP‐EGF‐H5E requires the presence of the Sm binding sequence on the siRNA for binding by the Sm proteins.

We then tested the stability of the reconstituted SmiRNP‐EGF‐H5E complex by incubating it at 4 °C and room temperature for 24 h and subsequently analyzing it by analytical size exclusion chromatography. The analytical size exclusion chromatography profiles of the complexes incubated at 4 °C and room temperature were similar to that of the freshly assembled SmiRNP‐EGF‐H5E control (Figure S3, Supporting Information), indicating that the SmiRNP‐EGF‐H5E complex is relatively stable post‐reconstitution at room and refrigerating temperatures.

To characterize the structure of SmiRNP‐EGF‐H5E, we performed negative stain electron microscopy of the SmiRNP‐EGF‐H5E complex. The negatively stained electron micrographs of SmiRNP‐EGF‐H5E presented a homogeneous spread of particles of ≈80 Å in diameter (Figure 2B). We picked 32001 particles and performed 2D classifications, which presented 2D averages of doughnut‐shaped complexes (top view) and top‐like tapered‐shaped complexes (side view) (Figure 2C; Figure S4, Supporting Information). These images resemble the expected shape of the U4 snRNP core (Figure 2C).^[^ ^7^, ^9^ ^]^ The diameter of the doughnuts is roughly 60 Å, which is similar to the diameter of the U4 snRNP core (PDB ID 4WZJ).^[^ ^7a^ ^]^ We then reconstructed a 3D density volume from the particles and docked the U4 snRNP core crystal structure model (PDB ID 4WZJ)^[^ ^7a^ ^]^ into the density volume (Figure 2D). Most of the U4 snRNP core could fit into the density volume (Figure 2D). However, the density corresponding to the EGF ligand, H5E, and part of the RNA stem loop was not observed, potentially due to the flexibility of these regions relative to the core. Nonetheless, the EM results confirmed that the purified ribonucleoprotein complex was homogeneous, with shapes and sizes resembling the U4 snRNP core.

### 2.2 SmiRNP‐EGF‐H5E Silences *KRAS* and Reduces Cell Viability in Colorectal Carcinoma Cells

**Figure 3:**
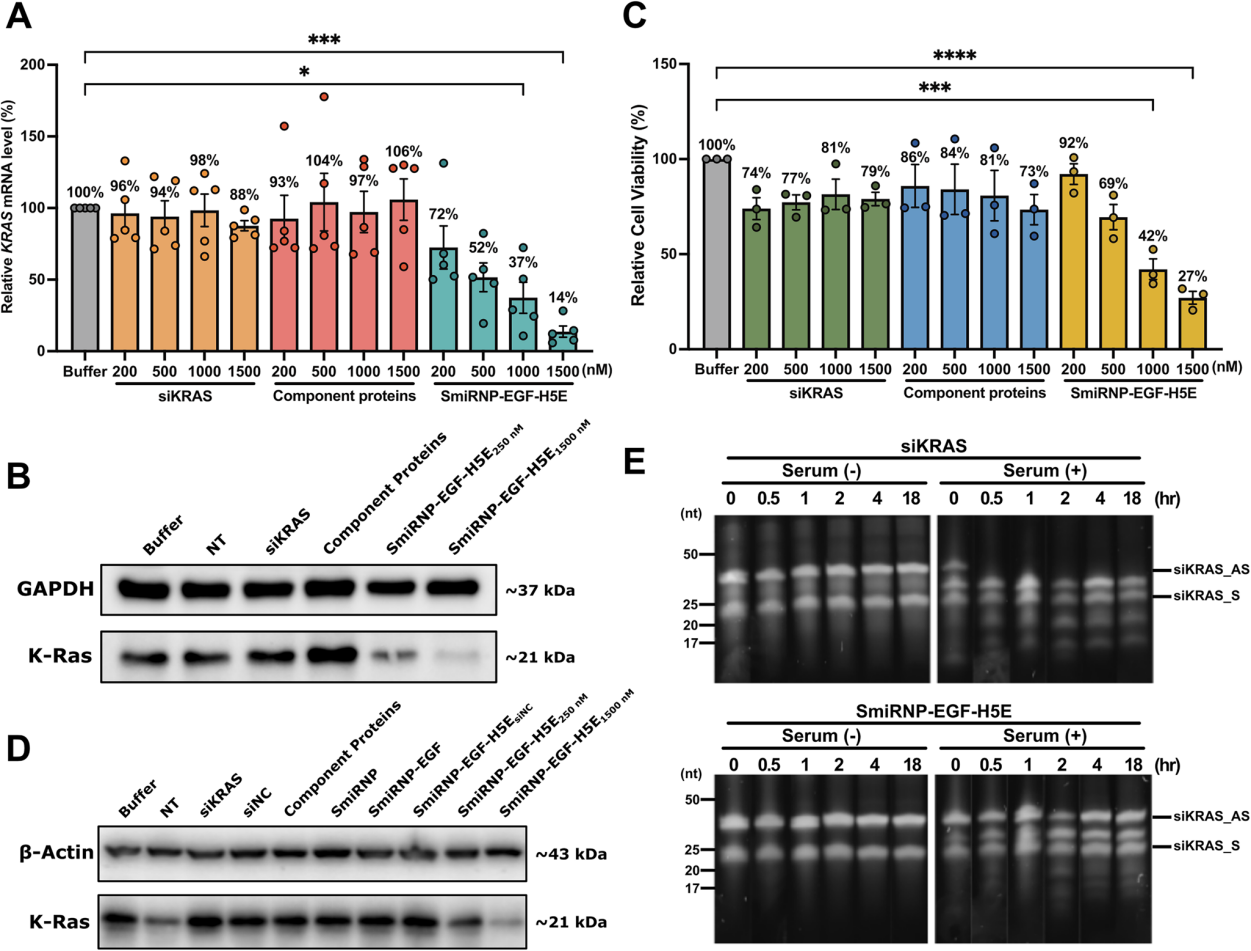
In vitro analyses of the gene silencing efficacy, cancer cell cytotoxicity, and stability of SmiRNP‐EGF‐H5E. A) Relative *KRAS* mRNA levels by RT‐qPCR of HCT116 cells incubated with buffer, siRNA, component proteins, and SmiRNP‐EGF‐H5E at concentrations of 200 to 1500 nm. Error bars represent ± SEM (*n* = 5). ^*^ *p* < 0.05, ^***^ *p* < 0.001 versus buffer group (One‐way ANOVA followed by Dunnett's test). B) Western blot analysis of K‐Ras protein levels in HCT116 cells with different treatments. NT, no treatment. C) MTT assay analysis of HCT116 cell viability treated with buffer, siRNA, component proteins, and SmiRNP‐EGF‐H5E at concentrations of 200 to 1500 nm. Error bars represent ± SEM (*n* = 3). ^***^ *p* < 0.001, ^****^ *p *< 0.0001 versus buffer group. D) Western blot analysis of K‐Ras protein levels for HCT116 cells when incubated with different variants of SmiRNPs. Note that the presence of both EGF ligand and H5E endosomal escape peptides is required for the reduction of K‐Ras levels. E) Comparison of siRNA stability as naked siRNA and when packaged in the SmiRNP‐EGF‐H5E complex in serum. siRNA_AS, siRNA antisense strand (36 nt); siRNA_S, siRNA sense strand (24 nt).

We next sought to demonstrate that the reconstituted SmiRNP‐EGF‐H5E complex can silence *KRAS* in HCT116 colorectal carcinoma cell line that carries the KRAS^G13D^ mutation and overexpresses the epidermal growth factor receptor (EGFR).^[^ ^15^ ^]^ We treated HCT116 cells with a range of SmiRNP‐EGF‐H5E concentrations (200 to 1500 nm). After 48 h, we determined the *KRAS* mRNA levels of the samples by reverse transcription quantitative real‐time PCR (RT‐qPCR). The RT‐qPCR results showed a SmiRNP‐EGF‐H5E concentration‐dependent decrease of *KRAS* mRNA levels relative to the buffer control (IC_50_ = 504 ± 23 nm) (**Figure** **3**A). At 1500 nm of SmiRNP‐EGF‐H5E, the *KRAS* levels were reduced to 14±4% relative to the buffer control. These results indicate that the SmiRNP fused EGF and H5E can lead to potent *KRAS* knockdown in HCT116 colorectal carcinoma cells.

To verify that the *KRAS* knockdown in HCT116 cells is reflected on the protein level, we performed western blot analysis. We observed a significant reduction of K‐Ras protein levels in HCT116 cells treated with 1500 nm of SmiRNP‐EGF‐H5E compared to the buffer control (Figure 3B), indicating that SmiRNP‐EGF‐H5E can lead to K‐Ras silencing in HCT116 cells at the protein level.

As HCT116 cells are K‐Ras‐dependent for viability,^[^ ^16^ ^]^ we wondered whether the SmiRNP‐EGF‐H5E complexes can reduce HCT116 cell viability by knocking down *KRAS*. We therefore performed MTT assays on HCT116 cells 72 h post‐treatment with a range of SmiRNP‐EGF‐H5E concentrations (200–1500 nm). The MTT assay results showed decreasing cell viability with increasing concentrations of SmiRNP‐EGF‐H5E (IC_50_ = 826 ± 13 nm) (Figure 3C). At the highest concentration of SmiRNP‐EGF‐H5E (1500 nm), HCT116 cell viability was reduced to 27 ± 3% relative to the buffer control. These results demonstrate the potential of SmiRNP‐EGF‐H5E as an anti‐cancer therapy against colorectal carcinoma by effectively reducing HCT116 cell viability.

### 2.3 Receptor‐Mediated Endocytosis and Endosomal Escape are Essential for Effective Gene Silencing

To probe the mechanisms of siRNA delivery by the SmiRNP‐EGF‐H5E complex, we performed fluorescence confocal microscopy studies to visualize the subcellular localization of a 5′‐FAM‐labelled siKRAS when delivered by SmiRNPs. We reconstituted three different versions of SmiRNPs carrying a fluorescent‐tagged 5′‐FAM‐siKRAS: 1) SmiRNP without EGF and H5E (FAM‐SmiRNP); 2) SmiRNP with EGF (FAM‐SmiRNP‐EGF); and 3) SmiRNP with both EGF and H5E (FAM‐SmiRNP‐EGF‐H5E) (Figure S1A–C, Supporting Information). We incubated each complex and just 5′‐FAM‐siKRAS with HCT116 cells for 6 and 18 h. Subsequently, we stained the lysosomes with LysoTracker Red and the nuclei with Hoechst 33342 and imaged them by confocal microscopy.

**Figure 4:**
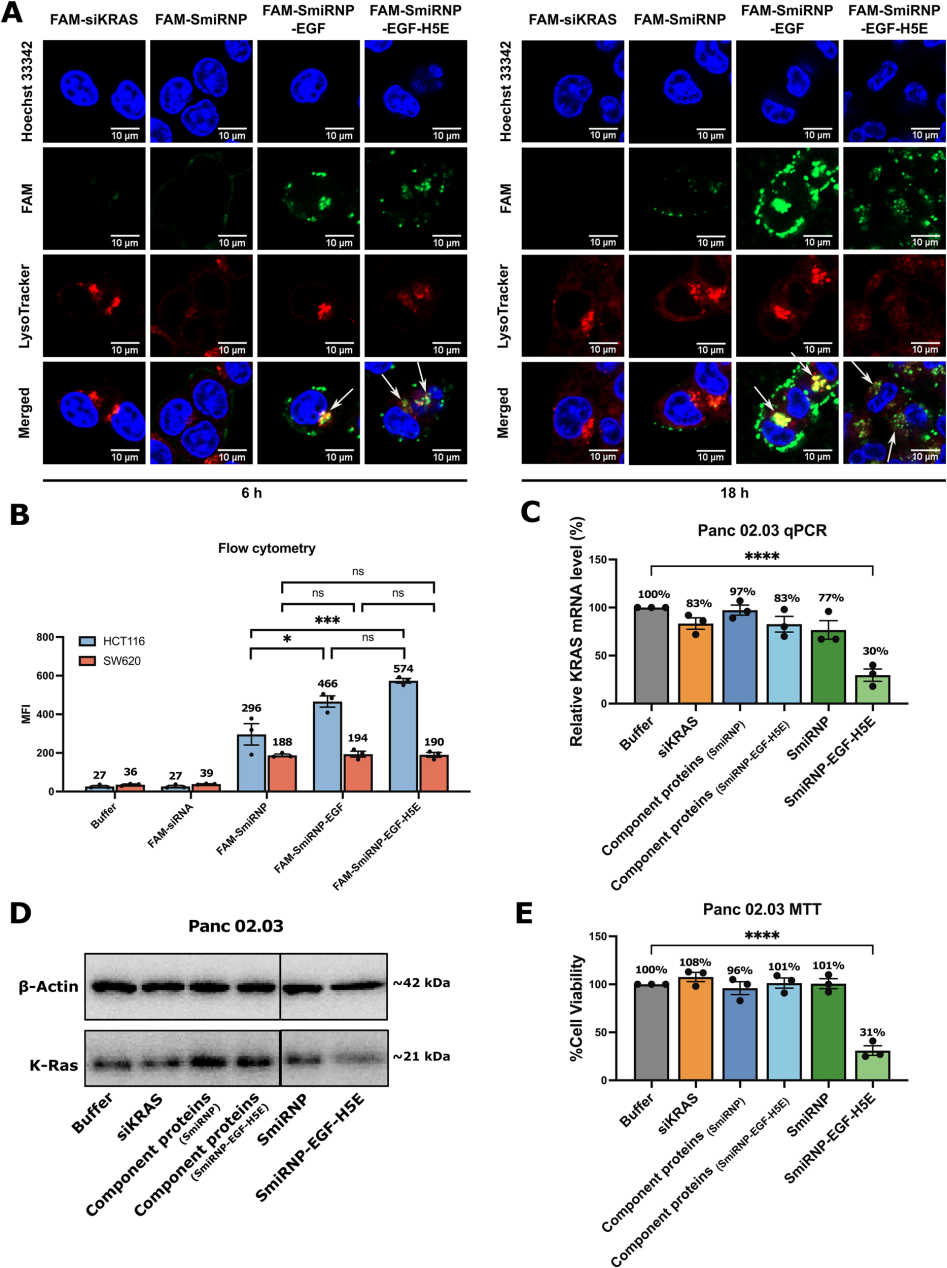
Cellular uptake of SmiRNP‐EGF‐H5E by EGFR‐mediated endocytosis. A) Co‐localization studies of SmiRNPs by confocal microscopy. HCT116 cells were incubated with 5′‐FAM labelled siKRAS (green) alone or various SmiRNPs containing a 5′‐FAM labelled siKRAS (green) for 6 and 18 h, stained with Hoeschst 33342 (blue) for the nuclei and Lysotracker Red for lysosomes (red) before being visualized by confocal microscopy. Note that cells treated with SmiRNPs containing EGF showed enhanced FAM signals (green) in the cells, indicating cellular uptake, while SmiRNP containing both EGF and H5E did not present co‐localization of siKRAS (green) and lysosome (red), suggesting endosomal escape. B) Flow cytometry analysis of uptake of SmiRNPs by EGFR‐positive HCT116 and EGFR‐null SW620 cells. Error bars represent ± SEM (*n* = 3). ns = not significant, ^*^ *p* < 0.05, ^***^ *p* < 0.001 (One‐way ANOVA followed by Tukey's test for each group). C) Relative *KRAS* mRNA levels by RT‐qPCR, D) relative K‐Ras protein levels by western blot analysis, and E) cell viability analysis by MTT assay of Panc 02.03 cells treated with buffer, siRNA, component proteins used to reconstitute SmiRNP, component proteins used to reconstitute SmiRNP‐EGF‐H5E, SmiRNP, and SmiRNP‐EGF‐H5E at a concentration of 1000 nM. Error bars represent ± SEM (*n* = 3). ^****^ *p *< 0.0001 versus buffer group (One‐way ANOVA followed by Dunnett's test).

For the 6 h time point, we observed FAM fluorescence signals in cells treated with FAM‐SmiRNP‐EGF and FAM‐SmiRNP‐EGF‐H5E, but not in cells treated with FAM‐SmiRNP and the FAM‐siKRAS alone (**Figure** **4**A), suggesting that fusion of the EGF ligand to the SmiRNP is essential for cell entry. Cells treated with FAM‐SmiRNP‐EGF showed some yellow signals (Figure 4A), suggesting that there was colocalization of FAM‐siKRAS (green) and the lysosome (red). However, cells treated with FAM‐SmiRNP‐EGF‐H5E showed largely distinct red and green signals near the nuclei (Figure 4A), indicating that the FAM‐siRNA did not localize in the lysosomes. For the 18 h time point, we observed a similar but more intense pattern of green and red fluorescent signal distributions in the FAM‐SmiRNP‐EGF and FAM‐SmiRNP‐EGF‐H5E samples (Figure 4A), suggesting that more FAM‐siKRAS entered the cells over the 12 h time difference. The FAM‐SmiRNP‐EGF‐treated cells showed intense green fluorescence signals around the edges of the cells and yellow signals near the nuclei, suggesting that FAM‐siKRAS is concentrated in vesicles near the plasma membrane (Figure 4A), likely due to endocytosis, and in the lysosomes. In contrast, cells treated with FAM‐SmiRNP‐EGF‐H5E for 18 h showed even more green signal puncta in the cytoplasm (Figure 4A), suggesting that FAM‐siRNA escaped the pathway that would have led to the lysosome.

We further confirmed that H5E is required for effective K‐Ras knockdown by western blot analysis. HCT116 cells incubated with SmiRNP‐EGF‐H5E exhibited lower K‐Ras levels relative to the buffer control (Figure 3D). However, cells treated with siKRAS, component proteins, SmiRNP (Figure S1D, Supporting Information), SmiRNP‐EGF (Figure S1E, Supporting Information), and an SmiRNP‐EGF‐H5E carrying an siRNA with a scrambled sequence of siKRAS as a negative control (siNC) (Figure S1F, Supporting Information), did not show a significant reduction in K‐Ras protein relative to the buffer control (Figure 3D). These western blot data corroborate the confocal microscopy findings, suggesting that while EGF is important for cell entry, H5E is required for endosomal escape of the siKRAS for effective gene silencing.

To quantify the uptake of FAM‐siKRAS by HCT116, we performed flow cytometry and compared their mean fluorescence intensities (MFI) of cells incubated with FAM‐siKRAS, FAM‐SmiRNP, FAM‐SmiRNP‐EGF, FAM‐SmiRNP‐EGF‐H5E, and buffer. The results showed that the FAM‐SmiRNP‐EGF and FAM‐SmiRNP‐EGF‐H5E samples showed higher MFI (470 ± 27 and 577 ± 12, respectively) than SmiRNP (without EGF) (298 ± 57) while buffer and siKRAS showed very minimal MFIs (27 ± 5 and 27 ± 5) (Figure 4B; Figure S5, Supporting Information). These results are consistent with the confocal microscopy data, supporting the finding that EGF aided uptake. To demonstrate that the absence of EGFR will not result in enhanced uptake of FAM‐siKRAS delivered by SmiRNPs fused with EGF, we performed a similar flow cytometry experiment on SW620 cells, which are EGFR‐null colorectal carcinoma cells, of which their lack of EGFR expression was verified by western blot (Figure S6A, Supporting Information). Flow cytometry analysis on SW620 showed that cells treated with FAM‐SmiRNP‐EGF and FAM‐SmiRNP‐EGF‐H5E had no significant differences in MFIs (187 ± 9 and 185 ± 13, respectively) compared to the cells treated with FAM‐SmiRNP (188 ± 6) (Figure 4B; Figure S5, Supporting Information), suggesting that the presence of EGF on the FAM‐SmiRNP‐EGF and FAM‐SmiRNP‐EGF‐H5E did not enhance uptake of the SmiRNP variants into the EGFR‐null SW620 cells. These results indicate that EGFR can aid the uptake of SmiRNP‐EGF and SmiRNP‐EGF‐H5E. We also further confirmed that SmiRNP‐EGF‐H5E did not cause significant knockdown of *KRAS* in SW620 by RT‐qPCR and western blot, and were not effective in reducing the cell viability of SW620 by MTT assay (Figure S6B–D, Supporting Information), further supporting the role of EGF as a ligand for endocytotic uptake of SmiRNP‐EGF‐H5E into HCT116.

To demonstrate that SmiRNP‐EGF‐H5E can be uptaken by another cell line that expresses EGFR, we tested SmiRNP‐EGF‐H5E on a pancreatic cancer cell line (Panc 02.03) that expresses EGFR (Figure S6A, Supporting Information) and carries a *KRAS* ^G12D^ mutation. RT‐qPCR analysis of *KRAS* mRNA levels showed that cells incubated with SmiRNP‐EGF‐H5E recorded *KRAS* mRNA levels of 30 ± 6% relative to those of the buffer control (Figure 4C). We confirmed by western blot that the K‐Ras protein had indeed been knocked down by SmiRNP‐EGF‐H5E (Figure 4D). MTT assays demonstrated that Panc 02.03 cell viability treated with SmiRNP‐EGF‐H5E was reduced to 31 ± 5% relative to buffer control (Figure 4E). These data indicate that SmiRNP‐EGF‐H5E can also silence *KRAS* and reduce the cell viability of another cancer cell line that is EGFR‐positive.

### 2.4 SmiRNP‐EGF‐H5E Protects siKRAS From Serum Nucleases

We then considered the stability of the siKRAS when delivered by SmiRNP‐EGF‐H5E via the bloodstream, which contains serum nucleases.^[^ ^3a^ ^]^ To investigate the extent of siRNA protection by SmiRNP‐EGF‐H5E, we separately incubated naked unmodified siKRAS and the SmiRNP‐EGF‐H5E complex in mouse serum and buffer, respectively, for 30 min, 1, 2, 4, and 18 h. After that, we analyzed the samples by denaturing Urea‐PAGE (Figure 3E). For the naked siKRAS and SmiRNP‐EGF‐H5E samples not exposed to serum, they showed largely intact bands corresponding to the sense and antisense strands of siKRAS (Figure 3E). For the naked siKRAS exposed to serum, the band corresponding to the antisense siKRAS strand cannot be observed from the 30 min time point onwards; lower molecular weight bands below the band corresponding to the sense strand could be observed, indicating RNA degradation (Figure 3E). The antisense siKRAS strand is more prone to degradation than the sense strand, likely because of the presence of a single‐stranded region corresponding to the Sm binding site, which is more exposed to exonuclease cleavage than the duplex region. On the other hand, the SmiRNP‐EGF‐H5E sample displayed intact sense and antisense strands on Urea‐PAGE throughout the 18 h time course (Figure 3E), suggesting that SmiRNP‐EGF‐H5E can provide significant protection to the siRNA from serum nucleases.

### 2.5 SmiRNP‐EGF‐H5E Suppresses HCT116 Tumor Growth in Mice

**Figure 5:**
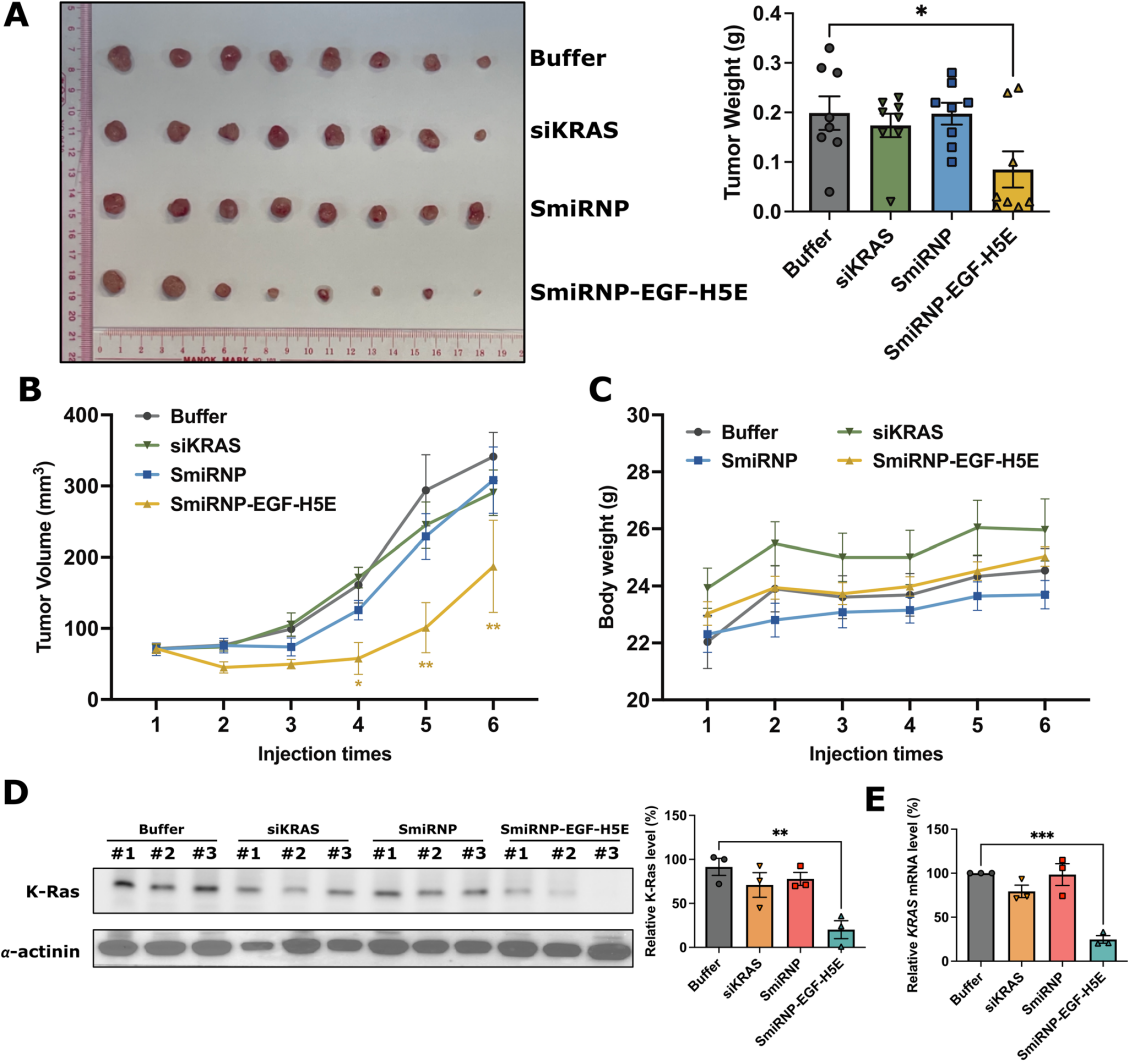
In vivo evaluation of SmiRNP‐EGF‐H5E in HCT116 xenograft‐bearing mice. A) Photos of the collected HCT116 xenografts (left) and weights of the tumors (right) are shown. B) Tumor volumes measured at each injection. C) Body weights of mice during the treatment period. In A) to C), error bars represent ± SEM (*n* = 8). ^*^p < 0.05, ^**^ *p *< 0.01, versus buffer group. D) Protein levels of K‐Ras in tumors. Representative blots (left) and statistical results (right) are shown. E) Relative *KRAS* mRNA levels by RT‐qPCR of tumors. In (D) and (E), error bars represent ± SEM (*n* = 3). ^**^ *p* < 0.01, ^***^ *p* < 0.001 versus buffer group (One‐way ANOVA followed by Dunnett's test).

We ultimately wanted to test the in vivo delivery of siKRAS and *KRAS* knockdown in tumors to demonstrate the potential clinical use of the SmiRNP delivery system. For this purpose, an HCT116 xenograft‐bearing mouse model was employed for in vivo investigations. The SmiRNP‐EGF‐H5E, or controls (buffer, siKRAS, and SmiRNP), was intravenously injected into the mice once every three days for a total of six injections. The mice were euthanized one day after the sixth injection, and the tumors were dissected, weighed, and photographed. The average tumor weight post‐sacrifice in the SmiRNP‐EGF‐H5E group (0.0850 ± 0.0364 g, 42.76% relative to buffer control) was markedly lower than that of the buffer group (0.1988 ± 0.0338 g), while the average tumor weights of the siKRAS (0.1738 ± 0.0237 g, 87.42% relative to buffer control) and SmiRNP (0.1975 ± 0.0221 g, 99.34% relative to buffer control) groups did not show significant differences from that of the buffer control (**Figure** **5**A). Notably, starting from the fourth injection, SmiRNP‐EGF‐H5E exhibited a significant inhibition of tumor growth, whereas the buffer, siKRAS, and SmiRNP groups displayed a consistent increase in tumor volume throughout the treatment duration (Figure 5B). These data strongly indicate that the SmiRNP‐EGF‐H5E complex could restrain tumor growth in vivo. The difference in tumor growth restraining efficacies between the SmiRNP and SmiRNP‐EGF‐H5E complexes suggests that EGF and H5E are required for effective delivery of siKRAS for *KRAS* gene silencing.

During the treatment period, no animal deaths or significant decrease in animal body weights (Figure 5C) were observed. No abnormalities were noted at necropsy or in terms of clinical signs; and no significant differences in food consumption were observed (Figure S7, Supporting Information). Additionally, after six dosing treatments, the levels of serum biochemical markers for liver and kidney function, including alanine transaminase (ALT), aspartate aminotransferase (AST), blood urea nitrogen (BUN), creatinine (Cre), remained similar with no significant differences across groups (Figure S8, Supporting Information). These results indicate that the SmiRNP complexes are biocompatible and show no apparent toxicity in mice.

The final injection for 3 mice from the siKRAS, SmiRNP, and SmiRNP‐EGF‐H5E groups contained a Cy5.5 fluorescent tag on the 5′‐end of the siKRAS (Figure S1G,H, Supporting Information) to track the biodistribution of siKRAS. The fluorescence of Cy5.5 was monitored using an Ivis Imaging System 1 h after intravenous injection. We observed that mice treated with SmiRNP‐EGF‐H5E exhibited fluorescence signals predominantly in the liver, whereas those treated with siKRAS or SmiRNP displayed fluorescence signals in both the liver and brain (Figure S9, Supporting Information). Although in vivo monitoring did not demonstrate an obvious accumulation of the Cy5.5‐labeled siRNA in the tumors (Figure S3, Supporting Information), western blot analysis revealed a significant reduction in K‐Ras protein levels in the tumors from the SmiRNP‐EGF‐H5E group, when compared to those from the buffer control group (Figure 5D). Tumor K‐Ras level reduction relative to that of buffer control was not observed in the siKRAS and SmiRNP groups (Figure 5D). These findings imply that siKRAS reached and effectively acted upon the tumor targets in the SmiRNP‐EGF‐H5E group, thereby achieving in vivo knockdown of *KRAS*.

Our findings indicate that SmiRNP‐EGF‐H5E effectively silences *KRAS* and exerts anti‐cancer effects without obvious toxicity in vivo. This proof‐of‐concept study demonstrates that the snRNPs can be remodeled as effective and biocompatible in vivo siRNA delivery agents.

### 2.6 SmiRNP can be Customized to Perform Gene Silencing by Targeting Other Receptors

**Figure 6:**
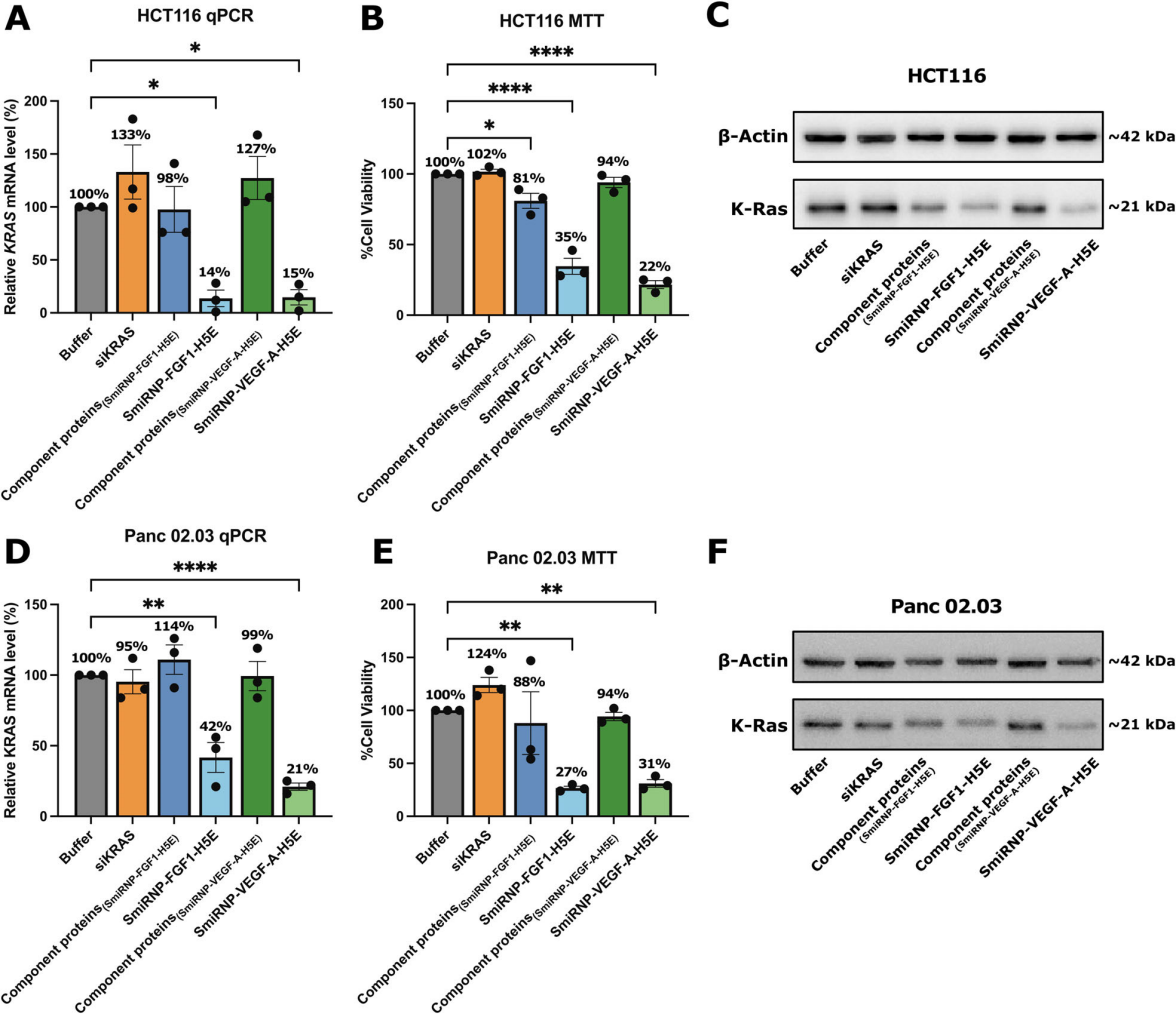
*KRAS* silencing by SmiRNP‐FGF1‐H5E and SmiRNP‐VEGF‐A‐H5E. A) Relative *KRAS* mRNA levels by RT‐qPCR, B) MTT assay analysis, and C) western blot analysis of HCT116 cells treated with buffer, siRNA, component proteins used to reconstitute SmiRNP‐FGF1‐H5E, SmiRNP‐FGF1‐H5E, component proteins used to reconstitute SmiRNP‐VEGF‐A‐H5E, and SmiRNP‐VEGF‐A‐H5E at a concentration of 1000 nM. D) Relative *KRAS* mRNA levels by RT‐qPCR, E) MTT assay analysis, and F) western blot analysis of Panc 02.03 cells treated with buffer, siRNA, component proteins used to reconstitute SmiRNP‐FGF1‐H5E, SmiRNP‐FGF1‐H5E, component proteins used to reconstitute SmiRNP‐VEGF‐A‐H5E, and SmiRNP‐VEGF‐A‐H5E at a concentration of 1000 nm. Error bars represent ± SEM (*n* = 3). ^*^ *p* < 0.05, ^**^ *p* < 0.01, ^****^ *p *< 0.0001 versus buffer group (One‐way ANOVA followed by Dunnett's test).

To demonstrate that the SmiRNP delivery system can be customized to target different cell surface receptors, we assembled two additional variants of SmiRNPs: 1) SmiRNP‐FGF1‐H5E (Figure S1I, Supporting Information), composed of the SmiRNP Sm core fused with fibroblast growth factor 1 (FGF1) and H5E, and carrying siKRAS; and 2) SmiRNP‐VEGF‐A‐H5E (Figure S1J, Supporting Information), composed the SmiRNP Sm core fused with vascular endothelial growth factor A (VEGF‐A) and H5E, and carrying siKRAS. These SmiRNPs were intended to respectively target the FGF receptors (FGFR) and the VEGF receptors (VEGFR) highly expressed in many cancer cell types. We then tested their abilities in silencing *KRAS* on HCT116 and Panc 02.03, both of which are K‐Ras‐dependent for viability. For HCT116, RT‐qPCR analysis of *KRAS* mRNA levels showed that incubation of 1000 nm SmiRNP‐FGF1‐H5E and SmiRNP‐VEGF‐A‐H5E reduced *KRAS* mRNA levels to 14 ± 8% and 15 ± 7%, respectively, relative to buffer control (**Figure** **6**A). MTT assay results also showed that SmiRNP‐FGF1‐H5E and SmiRNP‐VEGF‐A‐H5E reduced cell viability of HCT116 (35 ± 6% and 22 ± 3%, respectively, relative to buffer control) (Figure 6B). Western blot analysis of HCT116 also confirmed that SmiRNP‐FGF1‐H5E and SmiRNP‐VEGF‐A‐H5E reduced K‐Ras protein levels relative to the buffer control (Figure 6C). We also observed similar trends for the Panc 02.03 cells. SmiRNP‐FGF1‐H5E and SmiRNP‐VEGF‐A‐H5E reduced *KRAS* mRNA levels (42 ± 11% and 21 ± 3% relative to buffer control, respectively) in Panc 02.03 (Figure 6D), reduced Panc 02.03 cell viabilities (27±2% and 31 ± 4% relative to buffer, respectively) (Figure 6E), and reduced K‐Ras protein levels relative to buffer control (Figure 6F). These results showed that the SmiRNP delivery system is versatile and can be tailored for targeting different receptors for uptake and gene silencing. These studies also demonstrate that the SmiRNP system has the potential to be tailored for the delivery of siRNA to a wide range of different cell types.

## 3 Conclusion

We demonstrated that the U4 snRNP could be remodeled into an siRNA delivery agent in the form of a ribonucleoprotein complex. Adapting from established methods for snRNP in vitro reconstitution for structural biology studies,^[^ ^7^, ^9^, ^17^ ^]^ we reconstituted a modified Sm protein‐siRNA ribonucleoprotein complex (SmiRNP‐EGF‐H5E) that can effectively deliver siRNA into HCT116 colorectal carcinoma cells to silence the *KRAS* gene. Differing from previous attempts by others in using dsRBD proteins as a delivery agent,^[^ ^5^ ^]^ our investigations showed that the SmiRNP‐based delivery agent does not require chemical conjugations, such as ligand‐siRNA or polyethylene glycol (PEG)‐protein conjugations. The SmiRNP complexes, demonstrated in this study, only consist of unmodified siRNA and recombinant proteins. Future work could include testing the SmiRNP delivery system on modified siRNAs that have been shown to have better stability and efficacy in gene silencing compared to unmodified siRNAs. On the other hand, RNAi therapeutics can also address drug resistance, as encountered by therapies using small‐molecule K‐Ras inhibitors,^[^ ^18^ ^]^ through the alteration of the siRNA sequence to target K‐Ras with additional mutations or other downstream targets.

While the gene silencing efficacy of SmiRNP‐EGF‐H5E (IC_50_ = 504 nM) in HCT116 cells is still not comparable to that of lipofectamine transfection of siRNA,^[^ ^19^ ^]^ the SmiRNP delivery system that we reported here could target a non‐liver xenografted tumor and is not toxic, based on our mouse model studies. If the lack of toxicity holds true for humans, the SmiRNP delivery system has potential for clinical use given its ability to reach targets far from the liver. The potencies of SmiRNPs could be further improved, for instance, with the use of modified siRNAs. On the other hand, lipofectamine is known to be toxic and unsuitable for in vivo use.^[^ ^20^ ^]^ As proof‐of‐concept, we used nude mice for in vivo studies, but nude mice lack a full immune system, we were not able to perform thorough immunogenicity studies for the SmiRNPs. There is hope that the use of largely human‐based proteins for SmiRNP reconstitution will improve biocompatibility and reduce the risk of immunogenicity. These aspects have been reported through using human dsRBD proteins for siRNA delivery.^[^ ^5^ ^]^

Because SmiRNPs are heteromultimeric, we fused two different functional modules to the scaffold of the modified snRNP. As each Sm protein is unique, in theory, there are 12 more vacant sites for protein fusion, considering the possibilities of fusing polypeptides onto the N‐ and C‐termini of each Sm protein. Both termini of the Sm protein are exposed on the side of the Sm ring away from the siRNA duplex, and therefore protein fusion would not interfere with RNA binding (Figure S10), partly why we chose the SmiRNP system for siRNA delivery. In our prototype, we showed an example that we created a SmiRNP with a targeting module (EGF ligand) fused to SmD2 and an endosomal escape peptide (H5E) fused to SmB_1‐95_. These can be performed with ease by modifying the gene constructs for recombinant expression and purification of different versions of Sm proteins. We also showed that we can customize the SmiRNPs by switching the EGF ligand to either FGF1 or VEGF‐A, which also showed gene silencing efficacies in two different cancer cell types (colorectal carcinoma and pancreatic ductal adenocarcinoma). The combinations as a result of the 7 Sm proteins carrying a medley of fusion domains can be extensive. Different permutations of fusion domains can also be explored because the positions of each of the distinct Sm proteins relative to each other are fixed. Considering the modularity and customizability of this scaffold, its applications are likely to be broad for RNAi therapeutics delivery.

## 4 Experimental Section

### Expression and Purification of HIS‐SUMO‐SmD1/SmD2, His‐SUMO‐SmD1/SmD2‐EGF, His‐SUMO‐SmD1/SmD2‐FGF1 or His‐SUMO‐SmD1/SmD2‐VEGF‐A

SmD1 was cloned into the pET28a vector with an N‐terminal 6xHis‐SUMO tag. SmD2, SmD2‐EGF, SmD2‐FGF1, or SmD2‐VEGF‐A was cloned into the pCDFDuet‐1 (MCS2) vector. The SmD2‐EGF, SmD2‐FGF1, and SmD2‐VEGF‐A fusion proteins contain a linker with the amino acid sequence GGSGGS in between SmD2 and EGF, FGF1, or VEGF‐A. The plasmids encoding for SmD1 and SmD2, SmD2‐EGF, SmD2‐FGF1, or SmD2‐VEGF‐A were co‐transformed into *E. coli* BL21 (DE3) strain for co‐expression. The cells were first grown at 37 °C until they reached an OD_600_ of 0.6‐0.8 before being induced with a final concentration of 0.5 µm isopropyl β‐D‐thiogalactopyranoside (IPTG). Cell growth was then continued at 20 °C for 18 h before the cells were harvested by centrifugation.

For protein purification, cell pellets were resuspended in lysis buffer (20 mm Tris pH 7.5, 1 m NaCl, 10 mm imidazole, 5% glycerol, 10 mm β‐mercaptoethanol (βME) and complete Protease Inhibitor Cocktail (Roche)). The cells were lysed by sonication and centrifuged. The supernatant of the cell lysate was then loaded onto a 5 mL HisTrap FF column (Cytiva). The column was then washed with wash buffer (20 mm Tris pH 7.5, 1 m NaCl, 40 mm imidazole, 5% glycerol, 10 mm βME) and the proteins subsequently eluted with elution buffer (20 mm Tris pH 7.5, 1 m NaCl, 500 mm imidazole, 5% glycerol, 10 mm βME). The eluate was diluted with equal volume of Buffer A (20 mm HEPES pH 7.5, 5 mm dithiothreitol (DTT)) and subjected to further purification by heparin chromatography using a 5 mL HiTrap Heparin Column (Cytiva) on an ÄKTApure FPLC system (Cytiva) using a gradient Buffer A (20 mm HEPES pH 7.5, 5 mm DTT) to Buffer B (20 mm HEPES pH 7.5, 2 m NaCl, 5 mm DTT). The His‐SUMO‐tag was cleaved by ULP1 protease (protease to target protein ratio (w/w) of 1:20) and removed by nickel affinity chromatography. The purified protein was concentrated, flash frozen in liquid nitrogen, and stored at −80 °C.

### Expression and Purification of His‐SmD3/B_1‐95_ or His‐SmD3/B_1‐95_‐H5E

Plasmids pET28a_His‐SmD3 and pCDFDuet‐1_SmB_1‐95_ were provided by Liang Tong^[^ ^17^ ^]^ SmD3 was cloned into the pET28a vector with a N‐terminal 6xHis tag. SmB_1‐95_ or SmB_1‐95_‐H5E was cloned into the pCDFDuet‐1 (MCS2) vector. For protein expression, both proteins were co‐expressed in *E. coli* BL21 (DE3) strain as His‐SUMO‐SmD1/SmD2 as described above. The cells were first grown at 37 °C until it reached an OD_600_ of 0.6–0.8 before being induced with a final concentration of 0.5 µm IPTG. Cell growth was then continued at 20 °C for 18 h before the cells were harvested by centrifugation.

The protein purification protocols were adapted from a published report.^[^ ^17^ ^]^ Briefly, the cell pellets were resuspended in lysis buffer (20 mm Tris pH 7.5, 500 mm NaCl, 10 mm imidazole, 5% glycerol, 10 mm βME, and 17.8 µg mL^−1^ phenylmethylsulfonyl fluoride (PMSF)) and lysed by sonication as described above. The filtered lysate was then loaded onto a lysis buffer equilibrated 5 mL HisTrap FF column (Cytiva). The column was then washed with wash buffer (20 mm Tris pH 7.5, 500 mm NaCl, 40 mm imidazole, 5% glycerol, 10 mm βME) and subsequently eluted with elution buffer (20 mm Tris pH 7.5, 500 mm NaCl, 500 mm imidazole, 5% glycerol, 10 mm βME). The eluate was further purified by heparin chromatography using a 5 mL HiTrap Heparin Column (Cytiva) on an ÄKTApure FPLC system (Cytiva) using a gradient of Buffer A (20 mm HEPES pH 7.5, 5 mm DTT) to Buffer B (20 mm HEPES pH 7.5, 1 m NaCl, 5 mm DTT). The purified complex was concentrated, flash frozen, and stored at −80 °C.

### Expression and Purification of SmG‐His/SmE/SmF

Plasmids pET26b_SmG‐His and pCDFDuet‐1_SmE/SmF were provided by Liang Tong.^[^ ^17^ ^]^ SmG was cloned into the pET26b vector with a C‐terminal 6xHis tag. SmE and SmF were cloned into pCDFDuet‐1 vector (MCS1 and MCS2, respectively). For protein expression, all three proteins were co‐expressed in *E. coli* BL21 (DE3) strain and purified as SmD3/SmB_1‐95_ as described above.

### In vitro Reconstitution of SmiRNPs

siRNA (synthesized by Dharmacon, Horizon Discovery) was reannealed in DEPC‐treated water at 90 °C for 5 min followed by snap‐cooling on ice for 10 min. For the reconstitution of SmiRNPs, 1.5 molar equivalents of the component proteins were mixed with 1 molar equivalent of the siRNA in reconstitution buffer (20 mm HEPES pH 7.5, 650 mm NaCl, 5 mm EDTA, and 5 mm DTT). The reconstitution mixture was then incubated at 30 °C for 30 min, followed by 37 °C for 15 min, then cooled on ice. The SmiRNP complexes were then purified by size exclusion chromatography (Superdex 200 Increase 10/300 GL column or HiLoad 16/600 Superdex 200 pg, Cytiva) equilibrated with a buffer containing 20 mm HEPES pH 7.5, 500 mm NaCl, 10 mm EDTA, and 5 mm DTT. The purified complex was concentrated, flash frozen, and stored at −80 °C. The combination of the core proteins and siRNA for the different versions of the SmiRNP complexes can be found in Table S1 (Supporting Information).

### Negative Stain Electron Microscopy

The purified SmiRNP‐EGF‐H5E complex was diluted to 0.02 mg mL^−1^ and applied to a glow‐discharged carbon‐coated 300‐mesh copper TEM grid. The sample was stained with 2% (w/v) uranyl acetate before being loaded into a Talos L120C transmission electron microscope (ThermoFisher Scientific) operated at 120 kV. Data were acquired with a pixel size of 1.91 Å Px.^−1^ at a magnification of 45000 X by a 4k × 4k Ceta 16 M camera (ThermoFisher Scientific). The recorded data was processed by cryoSPARC v4.4.1.^[^ ^13^ ^]^ 32001 single particles were picked automatically and extracted with a box size of 100 Px. After an unbiased and reference‐free 2D classification, 26853 particles were selected for *ab‐initio* reconstruction of 3D EM density. The model of a crystal structure of the U4 snRNP core (PDB ID 4WZJ)^[^ ^7a^ ^]^ was docked into the EM density using PHENIX.DOCK_IN_MAP.^[^ ^14^ ^]^

### Cell Culture

Human colorectal carcinoma cell lines HCT116 and SW620, as well as human pancreatic adenocarcinoma epithelial cell line Panc 02.03, were purchased from the American Type Culture Collection (ATCC, USA). The HCT116 cells were cultured in McCoy's 5A medium (16600082, Gibco), the SW620 cells were cultured in high glucose Dulbecco's Modified Eagle Medium (DMEM) medium (12100061, Gibco), and the Panc 02.03 cells were cultured in RPMI 1640 medium (31800089, Gibco). All the media were supplemented with 10% fetal bovine serum (FBS, A5256701, Gibco) and 1x Antibiotic‐Antimycotic (15240062, Gibco). All cells were maintained at 37 °C in a humidified atmosphere of 5% CO_2_. The presence of mycoplasma is verified every 3 months using the MycoAlert PLUS Mycoplasma Detection Kit (#LT07‐703, Lonza, Basel, Switzerland).

### Quantification of KRAS mRNA Expression Levels Using RT‐qPCR

The HCT116, SW620, or Panc 02.03 cells were treated with indicated concentrations of SmiRNP, SmiRNP‐EGF‐H5E, SmiRNP‐FGF1‐H5E, SmiRNP‐VEGF‐A‐H5E, component proteins, siKRAS, or buffer. After 48 h of incubation, the cells were lysed with the TRIzol reagent (Invitrogen). Total RNA was extracted from cells using the Direct‐zol RNA Miniprep Kit (R2052, Zymo Research), following the manufacturer's protocol. 500 ng of each extracted RNA sample was reverse‐transcribed into cDNA using the PrimeScript RT Reagent Kit (RR037A, Takara). RT‐qPCR was performed on a StepOnePlus Real‐Time PCR instrument (Applied Biosystems) using iTaq Universal SYBR Green Supermix (1725124, Bio‐Rad). PCR reactions were performed in duplicates at 95 °C for 3 min, and 95 °C for 15 s, 60 °C for 1 min, for 40 cycles.

For in vivo studies, tumor tissues were homogenized in the TRIzol reagent using a tissue homogenizer (Servicebio, China). The homogenization was carried out in three cycles, each lasting 15 s at 60 Hz, with a 10 s pause between cycles. Total RNA was then extracted and reverse‐transcribed as described above. RT‐qPCR analyses were performed in duplicates with the same reaction and thermocycling conditions as described above using a ViiA 7 Real Time PCR System (Applied Biosystems).

The primer sequences corresponding to *KRAS* (amplicon length = 147 bp) were 5′‐GGCAAGAGTGCCTTGACGAT‐3′ (forward) and 5′‐CTCCTCTTGACCTGCTGTGT‐3′ (reverse). The primer sequences corresponding to the β‐actin gene (ACTB) (amplicon length = 198 bp) were 5′‐CCCAGCACAATGAAGATCAAG‐3′ (forward) and 5′‐GAAAGGGTGTAACGCAACTAAG‐3′ (reverse). The *KRAS* mRNA levels were normalized to the mRNA levels of *ACTB*, and the relative expression levels were calculated by the comparative CT (ΔΔCT) method.

### Analyses of K‐Ras Protein Levels by Immunoblotting (Western Blot)

The HCT116, SW620, or Panc 02.03 cells were treated with indicated concentrations of SmiRNP‐EGF‐H5E, SmiRNP‐FGF1‐H5E, SmiRNP‐VEGF‐A‐H5E, component proteins, siKRAS, or buffer. After 48 h of incubation, the cell pellets were harvested, washed with cold PBS, and lysed with the RIPA lysis buffer on ice for 30 min. The cell lysates were clarified by centrifugation at 15600 rcf at 4 °C for 15 min. The protein concentrations were determined using the Quick Start Bradford protein assay kit (5000205, Bio‐Rad). The samples were subjected to 10% SDS‐PAGE and transferred to polyvinylidene difluoride (PVDF) membranes (1620177, Bio‐Rad). The membranes were blocked with 5% blocking buffer (1X Tris‐buffered saline, 0.1% Tween 20, 5% (w/v) nonfat dry milk) for 1 h at room temperature. The membranes were then probed with diluted anti‐K‐Ras primary recombinant rabbit mAb (11H35L14, Invitrogen, 1:500), anti‐β‐actin (C4) primary mouse mAb (sc‐47778, Santa Cruz, 1:1000), and anti‐GAPDH (G‐9) primary mouse mAb (sc‐365062, Santa Cruz, 1:1000) at 4 °C overnight. Subsequently, the membranes were washed with Tris‐buffered saline supplemented with 0.1% Tween 20 (TBST) for 10 min for a total of three times. The membranes were then probed with corresponding diluted secondary antibodies using goat anti‐mouse IgG (1706515, Bio‐Rad, 1:3000) or goat anti‐rabbit IgG (1721011, Bio‐Rad, 1:3000) for 1 h at room temperature. The membranes were washed again with TBST buffer three times. Proteins were visualized by chemiluminescence using Immobilon Forte Western HRP Substrate (WBLUF0500, Millipore) with ChemiDoc imaging System (Bio‐Rad, USA). The protein bands were further analyzed using Image J.

For in vivo studies, tissue lysates were prepared using the RIPA lysis buffer, followed by homogenization. Western blot assays were performed as described above. The primary antibody for K‐Ras (11H35L14, Invitrogen, 1:500) was used to probe the membranes. The anti‐𝛼‐actinin (sc‐17829, Santa Cruz, 1:1000) was used as the loading control.

### Cell Viability Assay

The HCT116, SW620, or Panc 02.03 cells were separately treated with SmiRNP, component proteins, siRNA, or buffer at indicated concentrations in triplicate in a 96‐well plate. After 72 h of incubation, 10 µL of MTT (3‐[4,5‐dimethylthiazol‐2‐yl]‐2,5‐diphenylterazolium bromide) solution (5 mg mL^−1^) was added into each well and incubated for 2 h. The culture medium was removed, and 100 µL of DMSO was added into each well to dissolve the formed formazan crystals. The cell viabilities were determined by measuring the absorbance at 570 nm and referencing at 630 nm using a microplate spectrophotometer (BD Biosciences).

### Confocal Microscopy Imaging

HCT116 cells were seeded onto a µ‐Dish 35 mm Quad (80416, Ibidi). The cells were then treated with 150 nm FAM‐SmiRNP‐EGF‐H5E, FAM‐SmiRNP‐EGF, FAM‐SmiRNP or FAM‐siKRAS. After 6 h or 18 h of incubation, the culture medium was removed and stained with 50 nm LysoTracker Red DND‐99 (L7528, Thermo Fisher) and a drop of NucBlue Live ReadyProbe Reagent (R37605, Thermo Fisher) in PBS buffer at 37 °C for 5 min. Images of the cells were captured using a Nikon Eclipse Ti2 confocal laser‐scanning microscope.

### Flow Cytometry

HCT116 or SW620 cells were seeded in 24‐well plates at 70%–80% confluency and allowed to attach overnight. The cells were then serum‐starved for 4 h and treated with 250 nm FAM‐SmiRNP‐EGF‐H5E, FAM‐SmiRNP‐EGF, FAM‐SmiRNP or FAM‐siKRAS for 30 min. After incubation, the cells were washed three times with PBS, detached using trypsin, and centrifuged at 1500 rpm for 5 min. The pellets were resuspended in PBS, filtered through a 70 µm cell strainer (Corning, CLS431751; Merck), and analyzed using a BD FACSMelody Cell Sorter (BD Biosciences). 10000 events were collected for each sample. Data were processed with FlowJo v10 software (FlowJo LLC).

### Nuclease Protection Assay

SmiRNP‐EGF‐H5E or siKRAS (600 nm) were incubated in 50% mouse serum at 37 °C. For negative controls, the SmiRNP‐EGF‐H5E complex and siKRAS were incubated in PBS at 37 °C. Aliquots of 5 µL were collected at different time points (30 min, 1, 2, 4, and 18 h), and subjected to 12% Urea‐PAGE electrophoresis analysis stained with SYBR Gold (Thermo Fisher).

### Stability Analysis of SmiRNP‐EGF‐H5E

Freshly reconstituted and purified SmiRNP‐EGF‐H5E (600 mL; 1.5 mg/mL) at 4 °C was split into 3 equal portions. One of the portions was immediately loaded onto a Superdex 200 Increase 10/30 GL column (Cytiva) equilibrated with a buffer containing 20 mM HEPES pH 7.5, 500 mM NaCl, 10 mM EDTA, and 5 mM DTT for analysis. Another two vials were incubated at 4 °C and room temperature for 24 h respectively before being subjected to the same analytical size exclusion chromatography for analysis.

### SmiRNP‐EGF‐H5E Reconstitution Test with siKRAS and siKRAS (Sm^abs^)

Reconstituted samples (500 µL) for SmiRNP‐EGF‐H5E using siKRAS or siKRAS (Sm^abs^) (Dharmacon, Horizon Discovery) were first prepared as described in the methods for In vitro Reconstitution of SmiRNPs. Controls of the Sm protein mixture (SmD1/SmD2‐EGF, SmD3/SmB_1‐95_‐H5E, SmG/SmE/SmF) (30 µm of each protein subcomplex), siKRAS (20 µm), and siKRAS (Sm^abs^) (20 µm), were also subjected to steps in the in vitro reconstitution described above, with components in the absence being replaced with reconstitution buffer (20 mm HEPES pH 7.5, 650 mm NaCl, 5 mm EDTA, and 5 mm DTT). Subsequently, all 5 samples were then separately subjected to analytical size exclusion chromatography using a Superdex 200 Increase 10/30 GL column (Cytiva) equilibrated with a buffer containing 20 mm HEPES pH 7.5, 500 mm NaCl, 10 mm EDTA, and 5 mm DTT. Relevant fractions were further analyzed by 15% SDS‐PAGE electrophoresis and 12% Urea‐PAGE electrophoresis.

### Animal Experiments

Thirty‐two male 8‐week‐old BALB/c nu/nu mice with a body weight of 22 ± 2 g were purchased from GemPharmatech Co., Ltd (Jiangsu, China). All care and handling of animals were conducted with the approval of the Department of Health, Hong Kong (Licence No.: (21‐188) in DHHT&A/8/2/6 Pt.4). The animal experiment procedures were approved by the Committee on the Use of Human & Animal Subjects of the Hong Kong Baptist University.

HCT116 cells (5 × 10^6^) were suspended in 0.1 mL of phosphate‐buffered saline (PBS) and inoculated subcutaneously into the back of each mouse. Five days after cell injection, the mice were randomly divided into four groups with 8 mice per group. They were then intravenously injected with either a control buffer, 6 mg kg^−1^ of siKRAS, 6 mg kg^−1^ of SmiRNP, or 6 mg kg^−1^ of SmiRNP‐EGF‐H5E once every three days for 18 consecutive days (a total of six injections per mouse). To assess the siRNA biodistribution, on the final (sixth) injection, three mice from each of the siKRAS, SmiRNP, and SmiRNP‐EGF‐H5E groups were injected with the respective Cy5.5‐labeled siKRAS agents. To monitor the toxicity of the complexes, general clinical observations were made, and mouse body weights were recorded at each injection. Tumor volumes were measured using a vernier caliper at each injection to assess tumor growth. At the end of the experimental period (one day after the sixth injection), blood was collected from the abdominal aorta of the mice under anesthesia using isoflurane inhalation (Induction: oxygen flow, 1 L min^−1^; isoflurane, 3%. Maintenance: oxygen flow, 0.3 L min^−1^; isoflurane, 1%). Sera were prepared for toxicity assays. Subsequently, the mice were euthanized by excessive anesthesia (inhaling 5% isoflurane), and the tumors from each mouse were dissected, weighed, and photographed. A gross necropsy was performed on all the dissected organs and tissues. Proteins from tumor tissues were extracted using the RIPA lysis buffer and examined by western blot analyses. RNA levels were measured by the real‐time quantitative polymerase chain reaction (RT‐qPCR).

### Statistical Analysis

All data, unless otherwise specified, are presented as mean ± SEM. Comparisons were performed using one‐way ANOVA followed by Dunnett's or Tukey's test (as specified) using the GraphPad Prism 10 software. *p* < 0.05 was regarded as statistically significant.

## Conflict of Interest

WSA is the inventor of patents 17/930,088 and 110109035. WSA is the founder and director of Nuplex Limited. NYT, XW, and ZLY are co‐founders and shareholders of Nuplex lImited. Other authors declare no conflict of interest.

## Supporting information



Supporting Information

## Data Availability

The data that support the findings of this study are available from the corresponding author upon reasonable request.
