# Supplementary material for: Development of a Modular Ribonucleoprotein Complex as a General Strategy to Deliver RNAi Therapeutics
Source: Adv Healthc Mater. 2025 Jul 23;14(25):e03281. doi: 10.1002/adhm.202503281 (PMC12477566; doi:10.1002/adhm.202503281)
Supplement: Supplementary file 1 — Supporting Information [file ADHM-14-0-s001.pdf]

ADVANCED HEALTHCARE MATERIALS

Supporting Information

for *Adv. Healthcare Mater.*, DOI 10.1002/adhm.202503281

Development of a Modular Ribonucleoprotein Complex as a General Strategy to Deliver RNAi Therapeutics

Nok Yin Tam, Xiaoqi Wang, Grace Chung Yan Chan, Wai Po Kong, Wai Yin Chau, Xiuqiong Fu, Kwok-Yin Wong, Hong Lok Lung, Zhi-Ling Yu and Wei Shen Aik**

Supplementary Information

Title: Development of a modular ribonucleoprotein complex as a general strategy to deliver RNAi therapeutics

Nok Yin Tam[1]†, Xiaoqi Wang[2]†, Grace Chung Yan Chan[1,2], Wai Po Kong[3], Wai Yin Chau[1], Xiuqiong Fu[2], Kwok-Yin Wong[3], Hong Lok Lung[1], Zhi-Ling Yu[2]*, Wei Shen Aik[1]*

*Corresponding authors. Email: aikweishen@hkbu.edu.hk (W.S.A.); zlyu@hkbu.edu.hk (Z.L.Y.)

The PDF file includes:

Figures S1 to S10
Tables S1 to S2

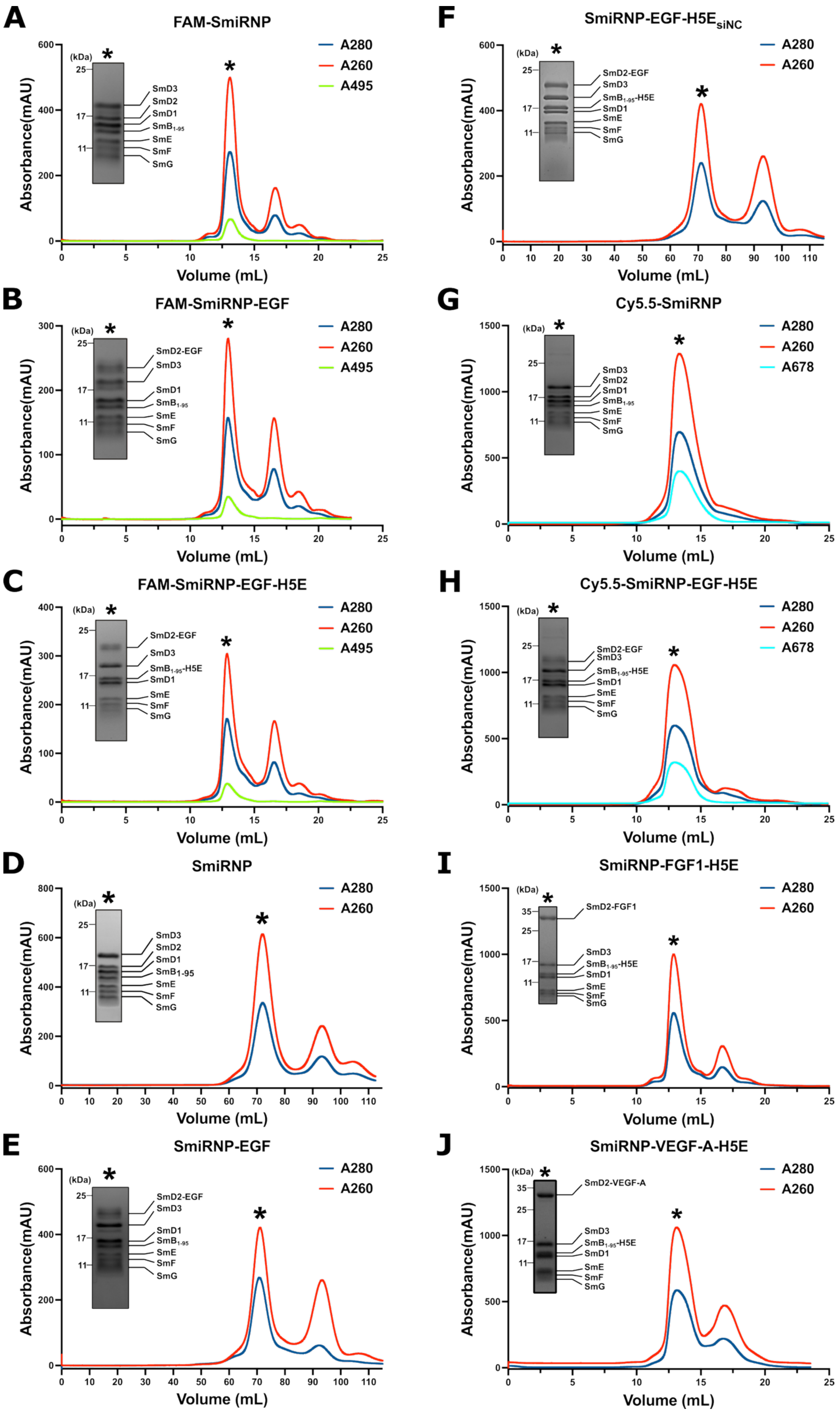
A
FAM-SmiRNP
A280
A260
A495
SmD3
SmD2
SmD1
SmB$_{1-95}$
SmE
SmF
SmG
B
FAM-SmiRNP-EGF
A280
A260
A495
SmD2-EGF
SmD3
SmD1
SmB$_{1-95}$
SmE
SmF
SmG
C
FAM-SmiRNP-EGF-H5E
A280
A260
A495
SmD2-EGF
SmD3
SmB$_{1-95}$-H5E
SmD1
SmE
SmF
SmG
D
SmiRNP
A280
A260
SmD3
SmD2
SmD1
SmB1-95
SmE
SmF
SmG
E
SmiRNP-EGF
A280
A260
SmD2-EGF
SmD3
SmD1
SmB$_{1-95}$
SmE
SmF
SmG
F
SmiRNP-EGF-H5E$_{siNC}$
A280
A260
SmD2-EGF
SmD3
SmB$_{1-95}$-H5E
SmD1
SmE
SmF
SmG
G
Cy5.5-SmiRNP
A280
A260
A678
SmD3
SmD2
SmD1
SmB$_{1-95}$
SmE
SmF
SmG
H
Cy5.5-SmiRNP-EGF-H5E
A280
A260
A678
SmD2-EGF
SmD3
SmB$_{1-95}$-H5E
SmD1
SmE
SmF
SmG
I
SmiRNP-FGF1-H5E
A280
A260
SmD2-FGF1
SmD3
SmB$_{1-95}$-H5E
SmD1
SmE
SmF
SmG
J
SmiRNP-VEGF-A-H5E
A280
A260
SmD2-VEGF-A
SmD3
SmB$_{1-95}$-H5E
SmD1
SmE
SmF
SmG
(kDa)
Absorbance(mAU)
Volume (mL)

Figure S1. *In vitro* reconstitution of SmiRNP complexes. Size exclusion chromatography profiles and SDS-PAGE analyses of the major peak (*) of the *in vitro* reconstituted (A) FAM-SmiRNP, (B) FAM-SmiRNP-EGF, (C) FAM-SmiRNP-EGF-H5E, (D) SmiRNP, (E) SmiRNP-EGF, (F) SmiRNP-EGF-H5E$_{siNC}$, (G) Cy5.5-SmiRNP, (H) Cy5.5-SmiRNP-EGF-H5E, (I) SmiRNP-FGF1-H5E and (J) SmiRNP-VEGF-A-H5E. Red line, 260 nm wavelength absorbance; blue line, 280 nm wavelength absorbance; cyan line, 678 nm wavelength absorbance; green line, 495 nm wavelength absorbance.

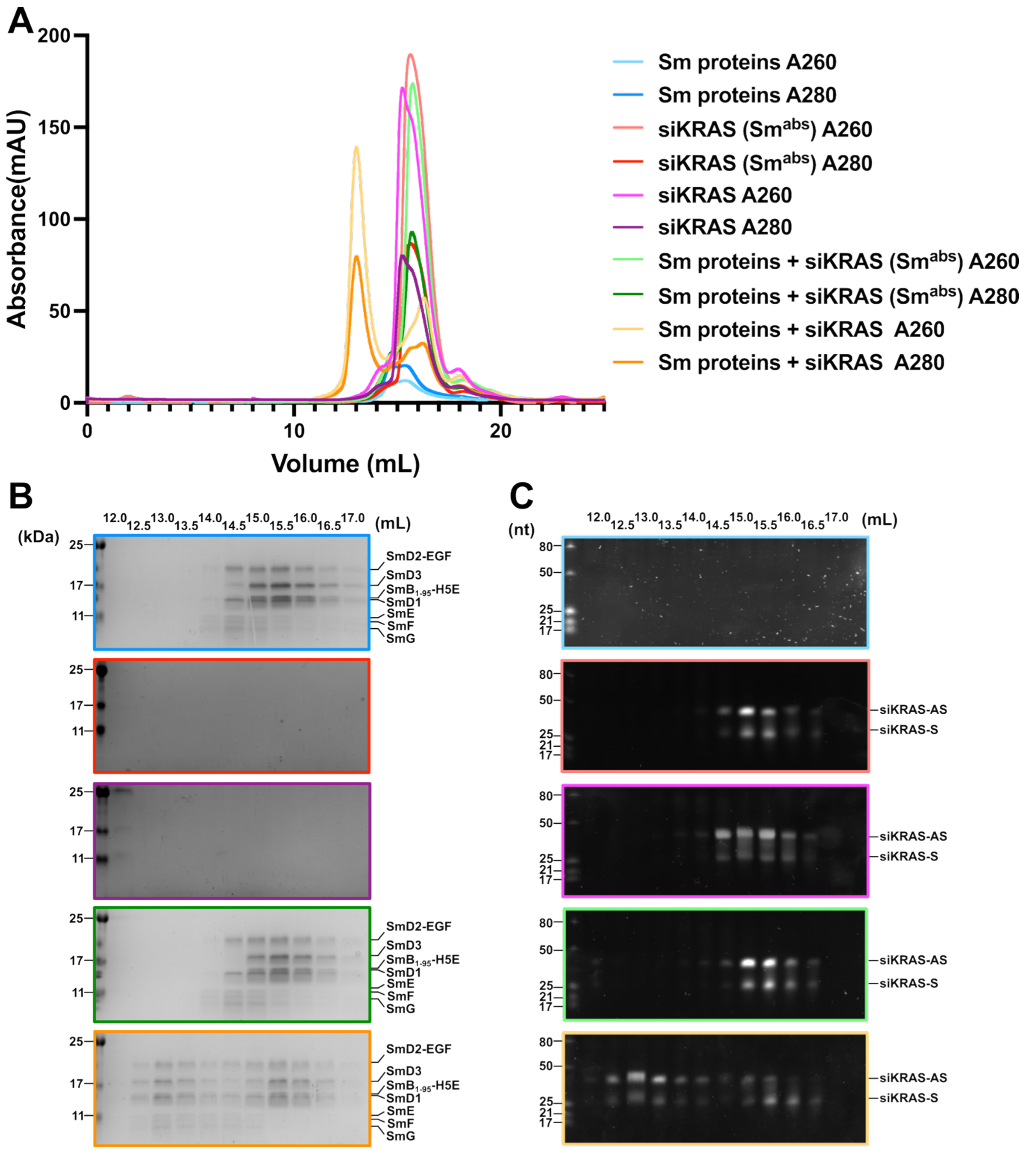

Figure S2. *In vitro* reconstitution of SmiRNP-EGF-H5E requires the Sm binding site on the siRNA. (A) Superdex 200 Increase analytical size exclusion chromatography overlaid 260 nm and 280 nm UV chromatograms of Sm proteins used for reconstitution (SmD1/SmD2-EGF, SmD3/SmB$_{1-95}$-H5E, SmG/SmE/SmF), siKRAS with the U4 Sm binding site replaced with poly-A's (Sm^{abs}), siKRAS, Sm proteins mixed with siKRAS (Sm^{abs}), and Sm proteins mixed with siKRAS. (B) SDS-PAGE of fractions from the analytical size exclusion chromatographies shown in (A). (C) Urea-PAGE of fractions from the analytical size exclusion chromatographies shown in (A).

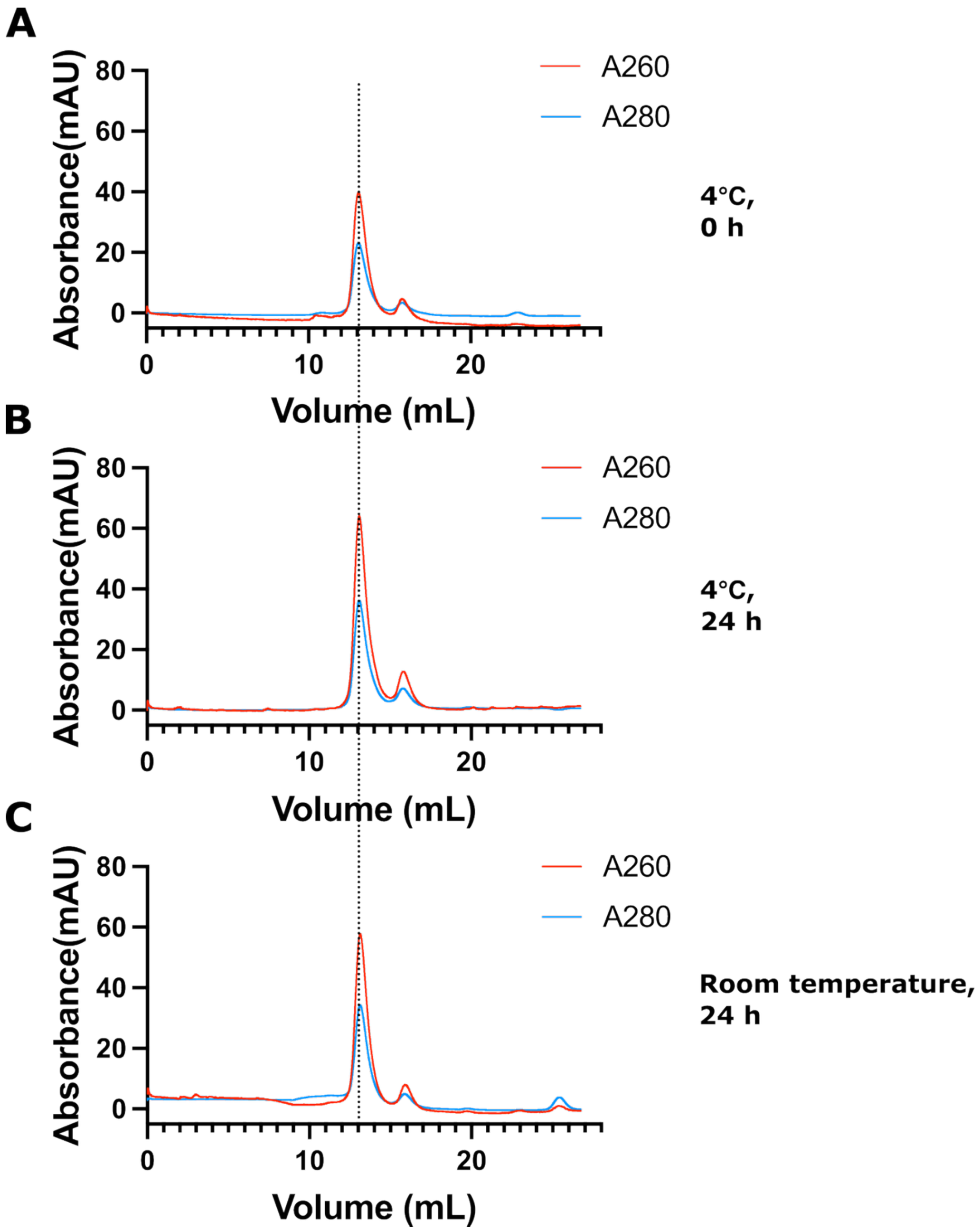

Figure S3. Stability analysis of SmiRNP-EGF-H5E. Superdex 200 Increase analytical size exclusion UV (260 nm and 280 nm) chromatograms of SmiRNP-EGF-H5E (A) freshly assembled and purified at 4 °C, (B) after incubation at 4 °C for 24 h, (C) after incubation at room temperature for 24 h.

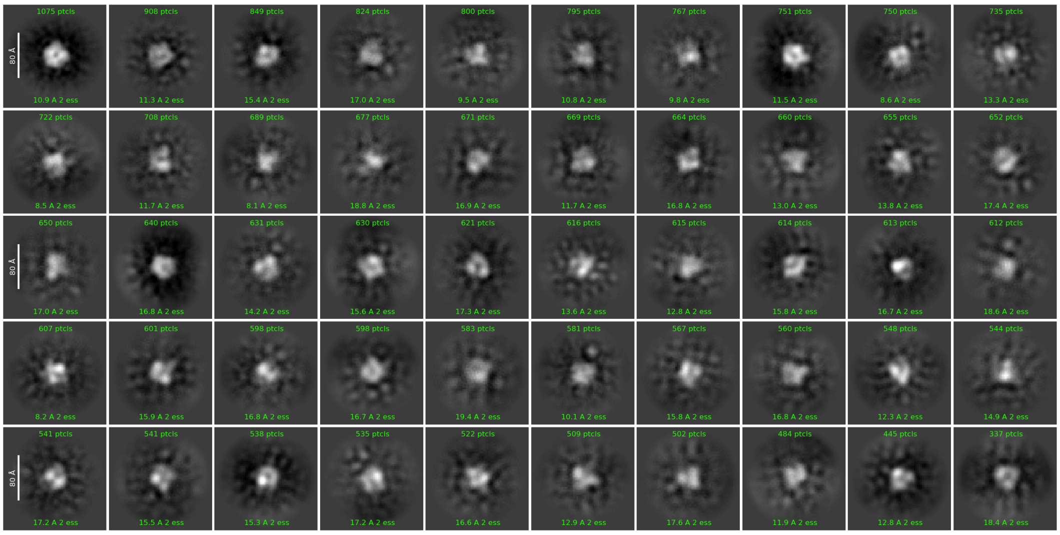

Figure S4. 2D class averages of SmiRNP-EGF-H5E by negative stain electron microscopy. Image generated by cryoSPARC v4.4.1[1]. 80 Å scale bars can be found on the far left.

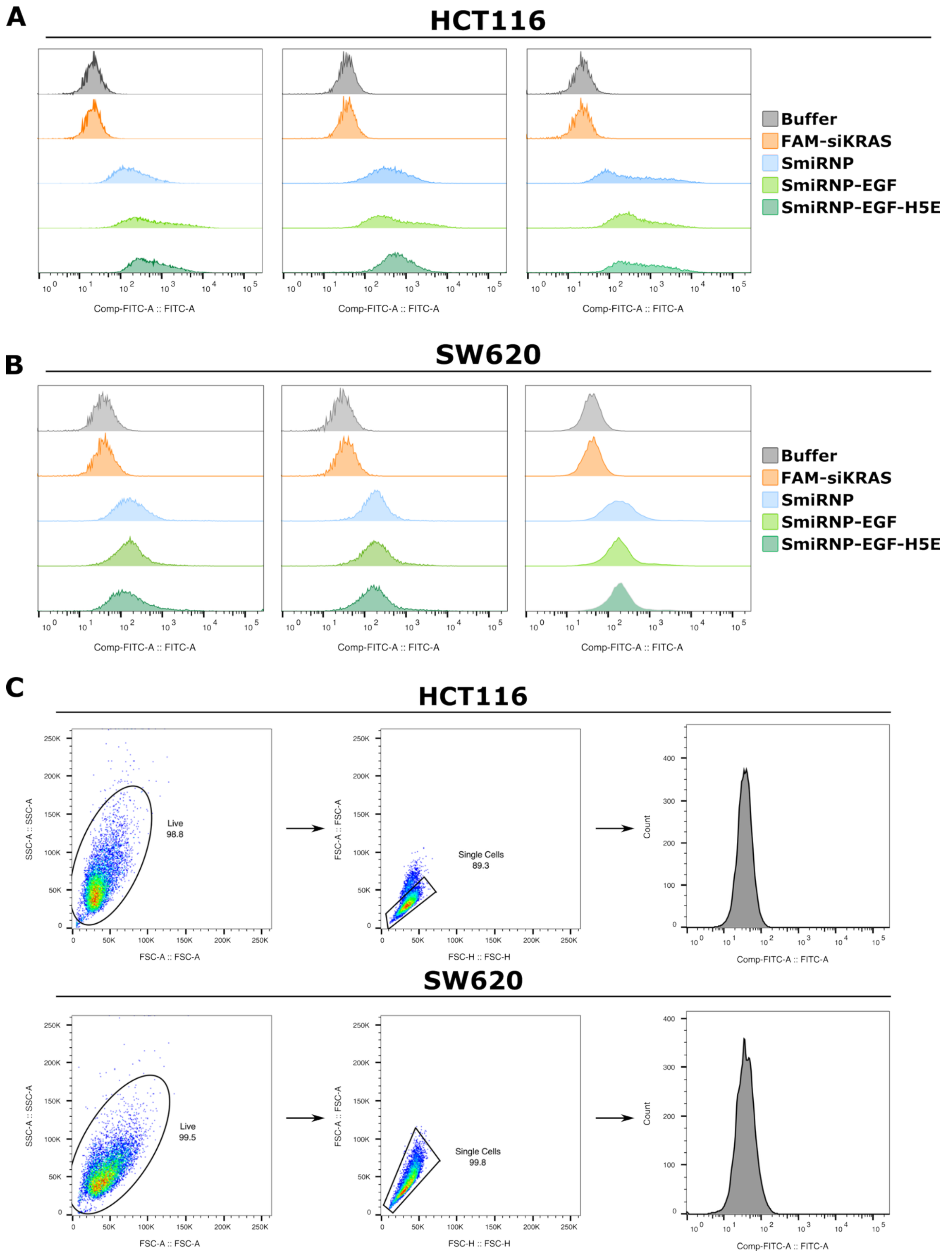

Figure S5. Flow cytometric quantification of FAM-siKRAS uptake in EGFR-positive HCT116 and EGFR-null SW620 cells. (A, B) Overlaid histograms of flow cytometry analysis showing FAM-siKRAS signal intensities in (A) HCT116 and (B) SW620 cells. A rightward shift in the fluorescence peak indicates increased intracellular uptake of FAM-siKRAS. Data are from three independent experiments. (C) Flow cytometry gating strategy for the uptake of FAM-siKRAS. Live cells were gated based on FSC-A versus SSC-A, followed by selection of single cells using FSC-A vs. FSC-H to exclude doublets. The mean fluorescence intensity

(MFI) in the FITC channel was quantified within the gated population to reflect the uptake of FAM-siKRAS in HCT116 (top) and SW620 (bottom) cells.

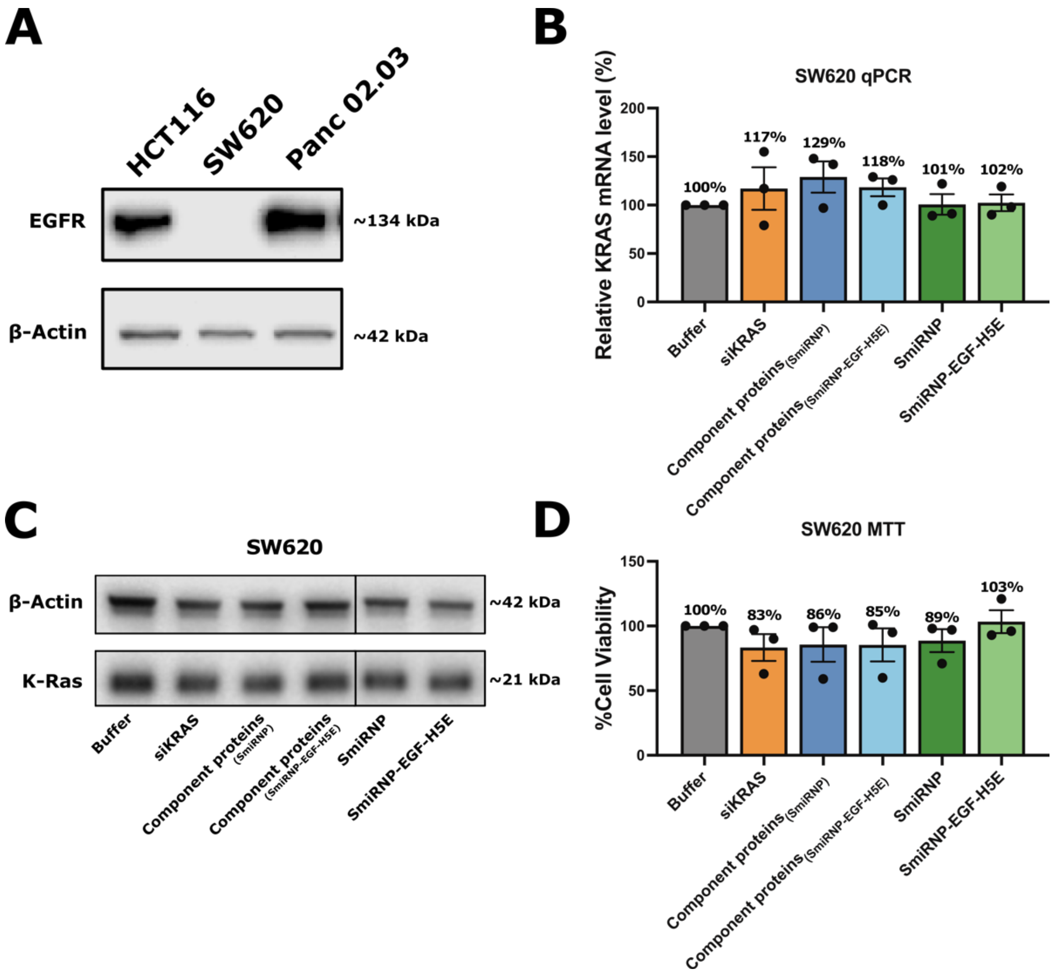

Figure S6. Investigations of *KRAS* silencing in EGFR-null SW620 cells by SmiRNP-EGF-H5E. (A) Western blot analysis of EGFR protein levels in HCT116, SW620, and Panc 02.03 cells. (B) Relative *KRAS* mRNA levels by RT-qPCR, (C) western blot analysis of K-Ras protein levels, and (D) MTT assay analysis of SW620 cells treated with buffer, siRNA, component proteins used to reconstitute SmiRNP, component proteins used to reconstitute SmiRNP-EGF-H5E, SmiRNP, and SmiRNP-EGF-H5E at a concentration of 1000 nM. Error bars represent ± SEM (n = 3). *$P < 0.05$, **$P < 0.01$, ***$P < 0.001$ vs buffer group (One-way ANOVA followed by Dunnett's test).

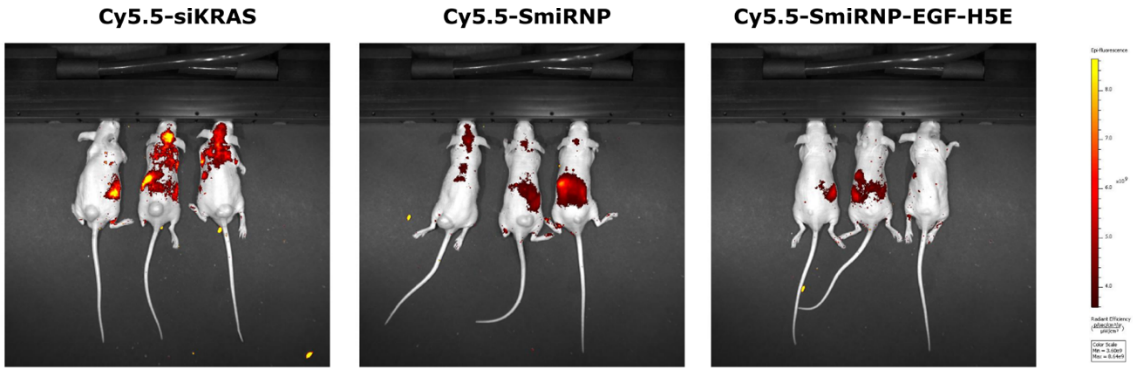

Figure S7. Biodistribution of siRNA in mice from the Cy5.5-siKRAS, Cy5.5-SmiRNP, and Cy5.5-SmiRNP-EGF-H5E groups. IVIS live imaging of three mice from each group (captured 1 h post-injection) is shown. A quantitative fluorescence intensity scale bar is displayed on the right.

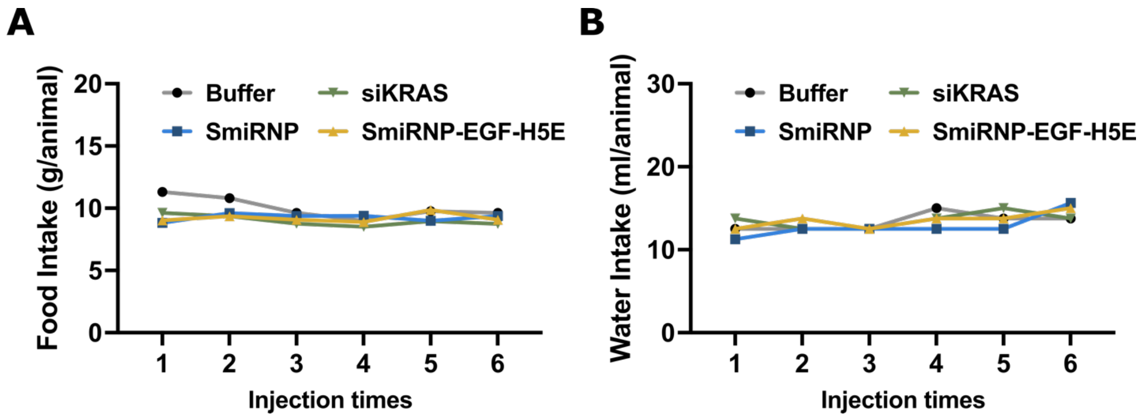

Figure S8. Water and food consumption of the mice. The total consumption of water and food of the mice in each group were recorded before every injection.

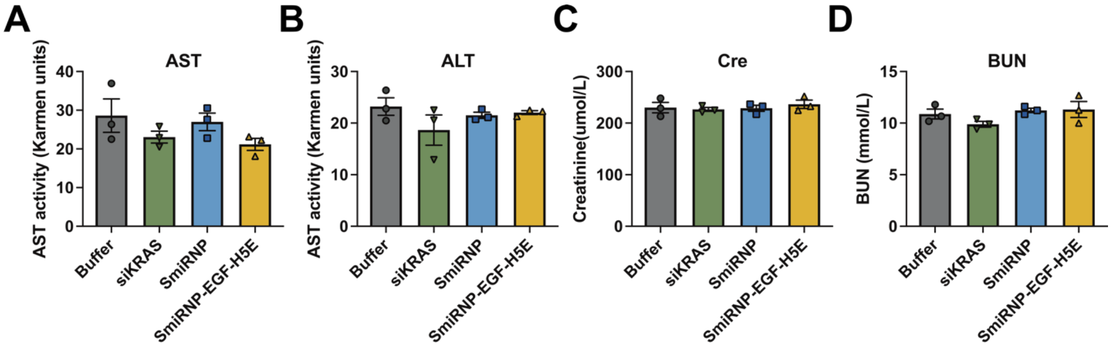

Figure S9. Levels of serum biomarkers showed no significant liver and kidney toxicity in HCT116 xenograft-bearing mice. Levels of (A) serum aspartate aminotransferase (AST), (B) alanine aminotransferase (ALT), (C) blood urea nitrogen (BUN), and (D) creatinine (Cre) in mice from each group are shown. Data were shown as mean ± SEM for three mice per group (n = 3).

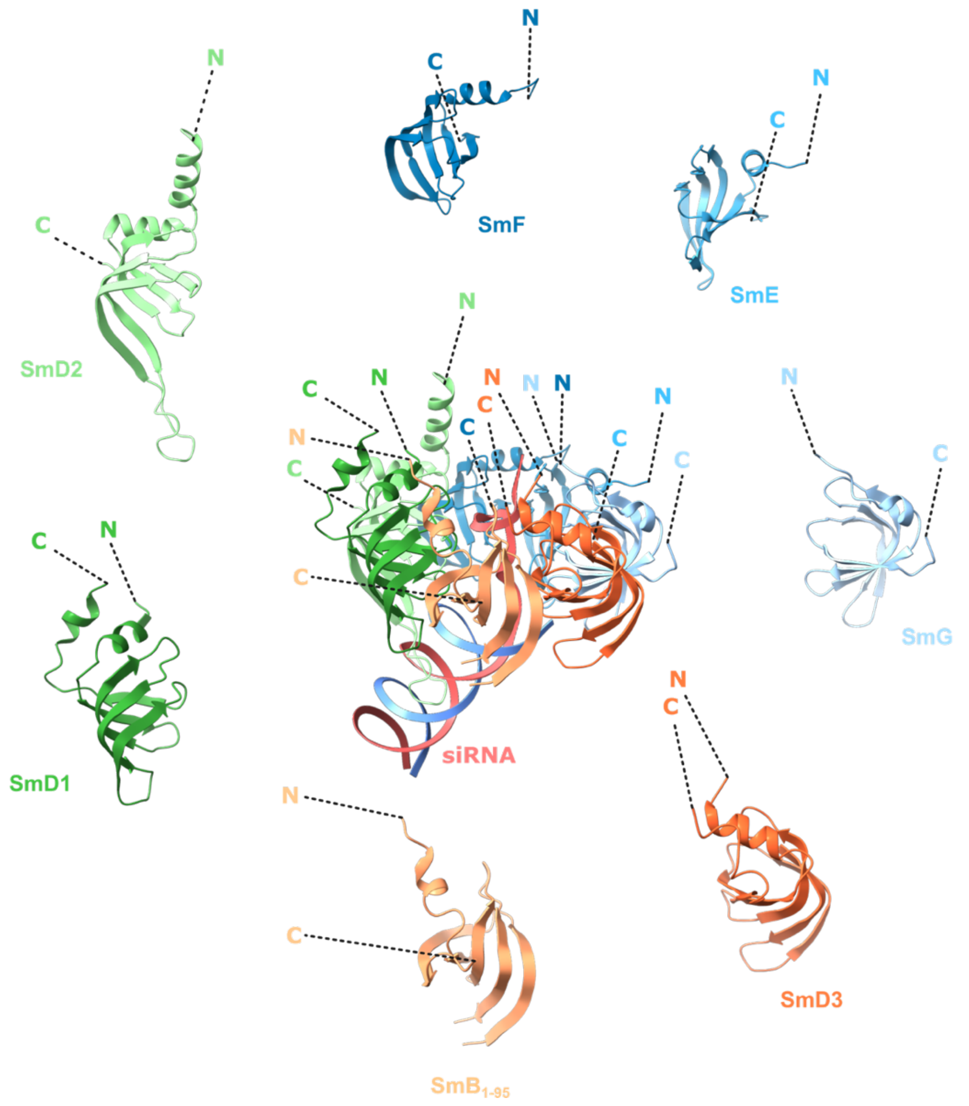

Figure S10. Locations of the N- and C-termini of the Sm proteins. Model of SmiRNP based on a partial model of the U4 snRNP core (PDB ID 4WZJ)[2] showing the positions of the N- and C-termini of the Sm proteins (labeled 'N' and 'C') are located on the side that is away from where the siRNA duplex is situated. There are 14 sites (N/C) available for protein fusion.

Table S1. Components of different variants of SmiRNPs used in this study.

Complex	Core Proteins	siRNA
SmiRNP-EGF-H5E	SmD1/SmD2-EGF	siKRAS
	SmG-His/SmE/SmF	
	His-SmD3/SmB$_{1-95}$-H5E	
SmiRNP-EGF	SmD1/SmD2-EGF	siKRAS
	SmG-His/SmE/SmF	
	His-SmD3/SmB$_{1-95}$	
SmiRNP	SmD1/SmD2	siKRAS
	SmG-His/SmE/SmF	
	His-SmD3/SmB$_{1-95}$	
SmiRNP-EGF-H5E$_{siNC}$	SmD1/SmD2-EGF	siNC
	SmG-His/SmE/SmF	
	His-SmD3/SmB$_{1-95}$-H5E	
SmiRNP-FGF1-H5E	SmD1/SmD2-FGF1	siKRAS
	SmG-His/SmE/SmF	
	His-SmD3/SmB$_{1-95}$-H5E	
SmiRNP-VEGF-A-H5E	SmD1/SmD2-VEGF-A	siKRAS
	SmG-His/SmE/SmF	
	His-SmD3/SmB$_{1-95}$-H5E	
FAM-SmiRNP-EGF-H5E	SmD1/SmD2-EGF	FAM-siKRAS
	SmG-His/SmE/SmF	
	His-SmD3/SmB$_{1-95}$-H5E	
FAM-SmiRNP-EGF	SmD1/SmD2-EGF	FAM-siKRAS
	SmG-His/SmE/SmF	
	His-SmD3/SmB$_{1-95}$	
FAM-SmiRNP	SmD1/SmD2	FAM-siKRAS
	SmG-His/SmE/SmF	
	His-SmD3/SmB$_{1-95}$	
Cy5.5-SmiRNP-EGF-H5E	SmD1/SmD2-EGF	Cy5.5-siKRAS
	SmG-His/SmE/SmF	
	His-SmD3/SmB$_{1-95}$-H5E	
Cy5.5-SmiRNP	SmD1/SmD2	Cy5.5-siKRAS
	SmG-His/SmE/SmF	
	His-SmD3/SmB$_{1-95}$	

Table S2. Sequences of the siRNA strands used in this study.

<table>
<tr><th>siRNA</th><th colspan="2">Sequence</th></tr>
<tr><td>siKRAS</td><td>Sense

Antisense</td><td>5' CCUUGACGAUACAGCUAAUUCAGA 3'
| | | | | | | | | | | | | | | | | | | | | | | |
3' UUGGAACUGCUAUGUCGAUUAAGUCUAGUUUUUAAC 5'</td></tr>
<tr><td>siNC</td><td>Sense

Antisense</td><td>5' GAAGCGUAUGCCACUACCUAUUAA 3'
| | | | | | | | | | | | | | | | | | | | | | | |
3' UUCUUCGCAUACGGUGAUGGAUAAUUAGUUUUUAAC 5'</td></tr>
<tr><td>FAM-siKRAS</td><td>Sense

Antisense</td><td>5' CCUUGACGAUACAGCUAAUUCAGA 3'
| | | | | | | | | | | | | | | | | | | | | | | |
3' UUGGAACUGCUAUGUCGAUUAAGUCUAGUUUUUAAC-FAM 5'</td></tr>
<tr><td>Cy5.5-siKRAS</td><td>Sense

Antisense</td><td>5' CCUUGACGAUACAGCUAAUUCAGA 3'
| | | | | | | | | | | | | | | | | | | | | | | |
3' UUGGAACUGCUAUGUCGAUUAAGUCUAGUUUUUAAC-Cy5.5 5'</td></tr>
</table>

Supporting references

[1] A. Punjani, J. L. Rubinstein, D. J. Fleet, M. A. Brubaker, "cryoSPARC: algorithms for rapid unsupervised cryo-EM structure determination" *Nat Methods* **2017**, *14*, 290-296 DOI: 10.1038/nmeth.4169.

[2] A. K. Leung, K. Nagai, J. Li, "Structure of the spliceosomal U4 snRNP core domain and its implication for snRNP biogenesis" *Nature* **2011**, *473*, 536-539 DOI: 10.1038/nature09956.
